# Artificial intelligence in breast cancer research: a systematic review and bibliometric analysis of emerging trends and future directions

**DOI:** 10.3389/fonc.2026.1800218

**Published:** 2026-09-28

**Authors:** Yathreb Bayan Mohamed, Hanaa ZainEldin, Shymaa G. Eladl, Hanaa A. Sayed, Mohamed Shehata, Mahmoud Badawy, Mostafa Elhosseini

**Affiliations:** 1 School of Public Health and Information Science, University of Louisville, Louisville, KY, United States; 2 Computers and Control Systems Engineering Department, Faculty of Engineering, Mansoura University, Mansoura, Egypt; 3 Higher Future Institute of Computer and Information Technology in Mansoura, Mansoura, Egypt; 4 Department of Computer Science, College of Computer Science and Engineering, Taibah University, Yanbu, Saudi Arabia; 5 Department of Computer Science, Faculty of Computers and Information, Assiut University, Assiut, Egypt; 6 Computer Science Program, James C. Bowling School of Business, Midway University, Midway, KY, United States; 7 Department of Computer Science and Information, Applied College, Taibah University, Medinah, Saudi Arabia; 8 Department of Information Systems, College of Computer Science and Engineering, Taibah University, Yanbu, Saudi Arabia

**Keywords:** artificial intelligence, breast cancer, breast datasets, explainability (XAI), hyperparameter optimization, imaging modalities mammograms, systematic review, vision transfomer

## Abstract

**Introduction:**

Breast cancer remains a leading cause of cancer incidence and mortality among women worldwide, with ongoing challenges in early detection, accurate diagnosis, prognosis estimation, and treatment-response assessment. Artificial intelligence (AI), including machine learning and deep learning, has rapidly advanced breast cancer research by enabling analysis of high-dimensional imaging, pathological, genomic, and clinical data. However, the rapid expansion of this literature has resulted in substantial fragmentation across clinical tasks, data modalities, algorithmic paradigms, and software ecosystems. This systematic review presents a comprehensive bibliometric analysis of AI-driven breast cancer research published recently.

**Methods:**

Using a multi-database, Scopus and Web of Science, as primary data sources, a systematic bibliometric framework integrating performance analysis, science mapping, and thematic evolution was employed. The analysis is guided by explicit research questions examining: (i) the evolution of AI applications across diagnosis, prognosis, and treatment-response prediction; (ii) the adoption of explainable AI and hyperparameter optimization practices; (iii) the datasets, software frameworks, and algorithms shaping the field; (iv) the role of mammography as a core modality; and (v) emerging research hotspots, including transformer-based architectures.

**Results and Discussion:**

Unlike prior bibliometric studies focused mainly on citation metrics, this work adopts a task-aware, methodologycentric perspective, offering actionable insights to support reproducible, interpretable, and clinically integrated AI systems for breast cancer care.

## 1 Introduction

Breast cancer is one of the most common and one of the deadliest diseases in the world from a female point of view. GLOBOCAN reports that there were approximately 2.3 million newly diagnosed breast cancer patients in 2020. This surpassed all other cancers, accounting for nearly 12% of all cancer net gain cases (1). In 2020, 685, 000 breast cancer fatalities were recorded, making it the fifth most fatal cancer, and the most fatal cancer for women (1). The American Cancer Society anticipates that there will be 310, 720 new cases of breast cancer and 42, 250 deaths from breast cancer in 2024. This illustrates the significant public health challenge that continues to exist, even after the improvement of breast cancer screening and treatment (2). Considerable improvement in breast cancer survivorship has been documented in high-income countries; 5-year survivals are greater than 90% after localized stage disease. Disparities in survivorship equality are prevalent in low-income countries, in rural regions, and for certain subtypes and stages of disease at diagnosis (2, 3). With the significant burden that breast cancer continues to cause in high-income and low-income countries, research investment is needed, particularly for the development of better risk stratification, early detection, accurate diagnosis, individualized treatment, and treatment strategies.

Recent years have shown an increased utilization of breast cancer studies and development combining artificial intelligence (AI) methods and technologies. Unique deep learning methods allow for detailed, complex pattern recognition, and have aided and vastly improved tumor detection and case outcome analysis. These methods have been applied to many forms of data, such as Mammogram, Ultrasound, and MRIs, alongside genomic and electronic health records (4). Recent studies for breast cancer related artificial intelligence have shifted toward clinically transparent and reproducible methods during recent years. Focusing on robust, XAI (explainable artificial intelligence) integration and trustable methods within the clinical workflows. The review studies have concentrated and noted the increase of multi methods of imaging, algorithm with the open data sets and the entire software ecosystems alongside the replicable deep learning frameworks for breast cancer detection and diagnosis. These noted and concentrated studies illustrate the current state of the art artificial intelligence breast cancer studies and research with clinical decision making and patient outcome improvement.

Research in the field of breast cancer is more needed, and investment in research in the field is vital. The world is in bad need of breast cancer risk, detection, accurate diagnosis, individualized treatment and treatment planning. The field of research in breast cancer is one of the research areas that is benefiting from the advancements in Artificial Intelligence Technologies. The ability of AI technologies to ‘learn’ and ‘follow’ complex tasks from high dimensional multi-structured data serves the field of breast cancer research a great deal in classifying, detecting, segmenting, and predicting outcomes.

Despite this progress, the rapidly expanding literature has become increasingly fragmented, spanning diverse imaging modalities, algorithmic paradigms, datasets, and clinical objectives. Understanding how these components interact and identifying dominant trends, emerging research fronts, and methodological gaps remain challenging. In this context, a systematic bibliometric-based analysis offers a robust framework for quantitatively mapping the intellectual structure, thematic evolution, and methodological priorities of AI-based breast cancer research. By synthesizing publication trends, algorithmic developments, explainability practices, dataset and software usage, and emerging architectures, such an analysis can provide critical insights to guide future research and support the development of clinically integrated AI systems for breast cancer care.

In response to the growing complexity and fragmentation of AI–driven breast cancer research, this systematic bibliometric-based analysis is structured around a set of focused research questions that systematically map methodological evolution, clinical priorities, and emerging technological directions. Specifically, the study addresses the following:

◼ RQ1. Field Evolution: How has AI-based breast cancer research evolved in recent years in terms of clinical tasks (diagnosis, prognosis, and treatment-response prediction), data modalities, and methodological paradigms?
◼ RQ2. Explainable AI and Trustworthiness: To what extent have explainable artificial intelligence (XAI) techniques been adopted in breast cancer research, and how do they contribute to model transparency, interpretability, and clinical trust?
◼ RQ3. Hyperparameter Optimization Practices: What trends characterize the use of hyperparameter optimization and model-tuning strategies in breast cancer AI studies, and what do they reveal about methodological rigor and reproducibility?
◼ RQ4. Datasets and Software Ecosystems: Which datasets, software frameworks, and computational tools are most frequently employed in breast cancer AI research, and how do these resources influence methodological choices and reproducibility?
◼ RQ5. Algorithmic Trends and Emerging Architectures: What algorithmic families dominate breast cancer AI research, and how rapidly are emerging architectures—particularly transformer-based models—gaining prominence?
◼ RQ6. Mammography as a Core Modality: What is the role and relative prominence of mammography within AI-driven breast cancer research, and how have mammography-based methodologies evolved over time?
◼ RQ7. Emerging Hotspots and Translational Potential: Which thematic hotspots exhibit the strongest growth and translational potential, and how can these insights inform the development of clinically integrated AI systems for breast cancer care?

Together, these research questions provide a structured foundation for the bibliometric-based analysis and the subsequent in-depth exploration of emerging hotspots presented in this study.

### 1.1 Key contributions of this bibliometric study

This study distinguishes itself from existing bibliometric-based analyses through the following key contributions:

- Task-aware bibliometric mapping systematically separating AI research in breast cancer across diagnosis, prognosis, and treatment-response prediction, rather than treating the field as a single unified domain.
- Methodology-centric analysis, incorporating dedicated evaluation of explainable artificial intelligence (XAI) adoption and hyperparameter optimization practices, which are rarely examined in prior bibliometric studies.
- Algorithm-level trend analysis, tracing the methodological evolution from classical machine learning to deep learning and emerging transformer-based architectures.
- Focused assessment of datasets and software ecosystems, providing insight into commonly used public datasets, computational frameworks, and toolchains that influence reproducibility and experimental design.
- In-depth exploration of emerging research hotspots, offering interpretive analysis of rapidly growing and clinically relevant research fronts beyond descriptive bibliometric reporting.

The following section describes the bibliometric methodology employed to systematically address these research questions, including data sources, search strategy, analytical tools, and the multi-layered analysis framework.

## 2 Bibliometric study

A systematic bibliometric search was conducted using a multi-database (Scopus and Web of Science) to identify scholarly publications about the application of artificial intelligence (AI) and deep learning techniques in breast cancer imaging.

### 2.1 Search strategy and data collection

Three independent search strategies were executed to ensure comprehensive coverage of literature. The first query combined breast cancer diagnostic terminology with deep learning and specific imaging modalities (“breast cancer detection” OR “breast cancer diagnosis”) AND (“deep learning” OR “CNN” OR “convolutional neural network” OR “vision transformer”) AND (mammography OR ultrasound OR MRI OR histopathology OR “whole slide image”), yielding 1209 records. The second query broadened the AI-related terminology (“breast cancer detection” OR “breast cancer diagnosis”) AND (“deep learning” OR “DL” OR “CNN” OR “convolutional neural network” OR “vision transformer” OR “AI” OR “Artificial Intelligence”) and identified 1923 records. The third query employed a more generalized formulation (“BREAST CANCER detection”) AND (“Deep learning” OR “DL” OR “AI” OR “Artificial intelligence”), resulting in an additional 1, 699 records. Collectively, the three searches produced a combined total of 4, 831 records.

Following automated and manual deduplication, 2744 duplicate entries were eliminated, leaving 2087 unique records for screening. The selection process was strictly followed by predefined inclusion and exclusion criteria. The inclusion criteria specified that publications must: (1) focus explicitly on breast cancer; (2) involve medical imaging modalities such as mammography, ultrasound, MRI, histopathology, or other diagnostic techniques; (3) use artificial intelligence, machine learning, deep learning, computer vision, or related methods in detection, diagnosis, classification, segmentation, or feature extraction; and (4) be published in English and indexed as indexed as article or review. All records passing the duplicate removal stage met these criteria, with no exclusions based solely on inclusion factors.

The exclusion criteria aimed to remove publications outside this study’s methodological scope. Studies were excluded if they: (1) focused mainly on therapeutic interventions like chemotherapy, radiotherapy, targeted agents, or treatment response modelling; (2) discussed prognosis, recurrence, or survival prediction without imaging components; (3) concentrated on genomic, proteomic, molecular, cellular, or biomarker analyses unrelated to imaging; or (4) examined cancer broadly without specific relevance to breast cancer. Papers relying only on traditional image processing techniques without machine learning or deep learning, or those presenting only clinical or statistical analyses without computational imaging models, were also excluded. (5) Conference papers, Book chapters, Books, Annual meetings, Abstracts, Dissertations, and Non-English papers are also excluded. This led to 608 records being discarded.

A systematic review was performed using the Scopus and Web of Science databases to identify scholarly publications on artificial intelligence (AI) and deep learning applications in breast cancer imaging. The Bibliometrix R package was employed to support bibliometric data processing and descriptive analysis, including dataset organization, frequency analysis, and statistical summarization of publication and citation data. To ensure comprehensive literature coverage, three independent search strategies were performed. The first query combined breast cancer diagnostic terminology with deep learning and imaging modalities, yielding 1209 records. The second query expanded the AI-related terminology and retrieved 1923 records. The third query employed a broader AI-based breast cancer detection formulation and identified an additional 1699 records. Collectively, the searches produced 4831 records.

Following automated and manual deduplication, 2744 duplicate records were removed, leaving 2087 unique publications for title and abstract screening. The study selection process was conducted according to the Preferred Reporting Items for Systematic Reviews and Meta-Analyses (PRISMA) framework, consisting of identification, screening, eligibility assessment, and final inclusion stages.

During the title and abstract screening stage, studies were excluded if they: (1) were unrelated to breast cancer; (2) did not involve imaging modalities such as mammography, ultrasound, MRI, histopathology, or related diagnostic imaging techniques; (3) did not apply artificial intelligence, machine learning, deep learning, computer vision, or related computational approaches; (4) focused primarily on treatment response, prognosis, recurrence, or survival prediction without imaging-based diagnostic analysis; (5) concentrated on genomic, molecular, or biomarker analyses unrelated to imaging; (6) lacked sufficient methodological or technical details relevant to the bibliometric scope of the study; (7) relied solely on conventional image processing techniques without AI or machine learning methodologies; or (8) were published in languages other than English. After screening, 1259 studies remained and were subjected to full-text eligibility assessment.

During the eligibility stage, full-text articles were carefully evaluated according to predefined inclusion and exclusion criteria. Studies were excluded if they are conference papers, conference reviews, book chapters, books, annual meeting abstracts, dissertations, and non-peer-reviewed publications were excluded during the eligibility assessment phase.

Following full-text eligibility evaluation, 608 publications satisfied all inclusion criteria and were included in the final bibliometric and science-mapping dataset, consisting exclusively of peer-reviewed journal articles and review papers published between 2020 and 2026. All bibliometric analyses, citation analyses, thematic evolution analyses, and VOSviewer-based science-mapping visualizations presented in this study were conducted using this final dataset of 608 eligible publications. The complete study selection process is summarized in the PRISMA workflow presented in **Figure 1**.

**Figure 1:**
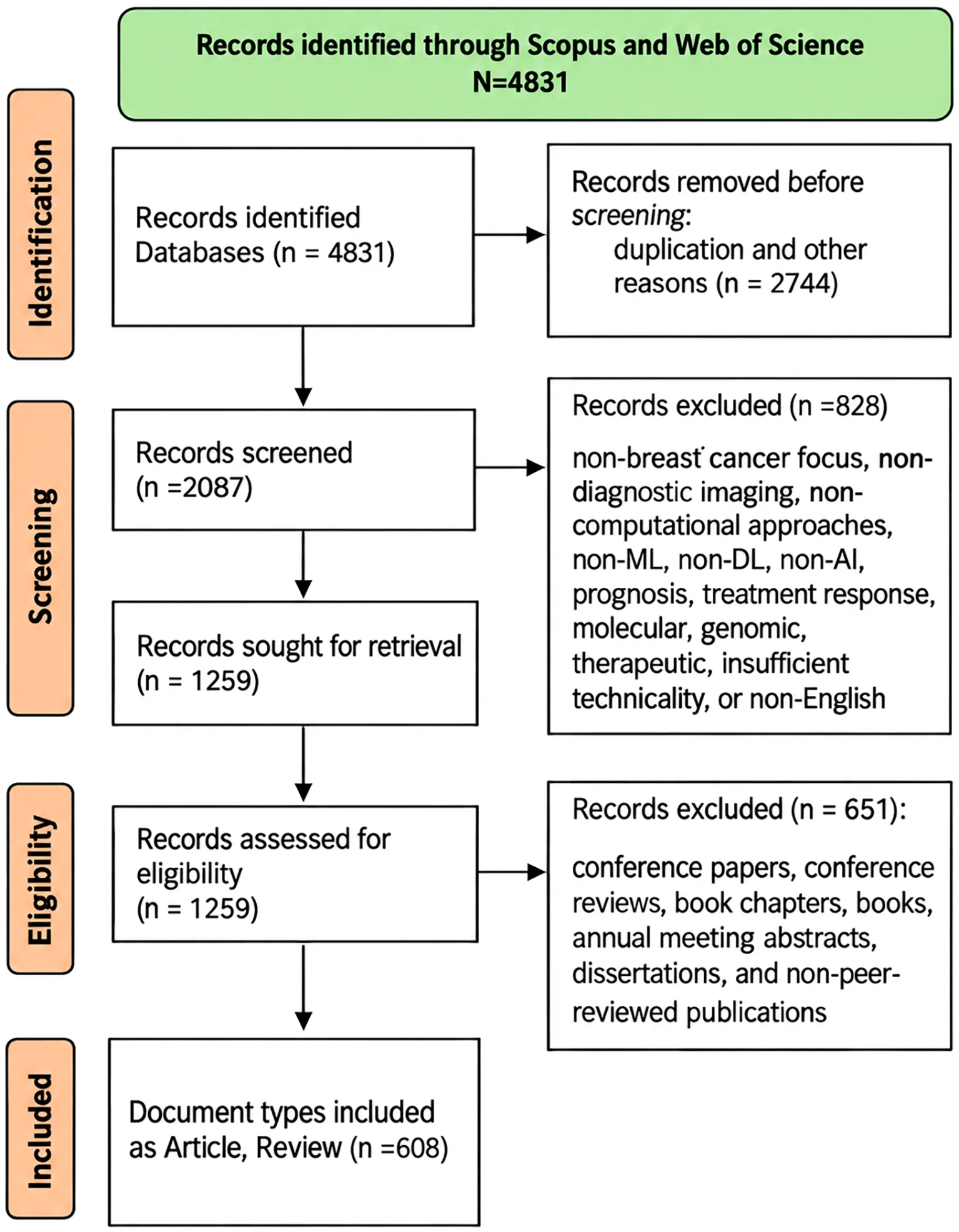
PRISMA workflow diagram.

This review was not prospectively registered in PROSPERO, the Open Science Framework (OSF), or any other public registry. The literature search, title and abstract screening, full-text eligibility assessment, and final study selection were conducted by a single reviewer using predefined inclusion and exclusion criteria. As the study was performed by a single reviewer, duplicate independent screening and formal disagreement resolution procedures were not applicable. The primary objective of this work was to combine a systematic literature review with bibliometric analysis to characterize research trends and emerging developments in artificial intelligence for breast cancer imaging.

### 2.2 recent trends in breast cancer research

The evolution of AI-based breast cancer research in recent years reveals a clear shift in focus across the three major clinical tasks diagnosis, prognosis, and treatment-response prediction with a strong dominance of diagnostic-oriented studies and a gradual expansion toward more comprehensive clinical decision support.

The analysis of the corpus (n = 608) in **Table 1** demonstrates that diagnosis remains the primary focus, accounting for 71.22% of all studies. Diagnostic AI systems encompass tasks such as detection, classification, segmentation, and screening, and are nearly universal in the literature. In particular, detection-related objectives appear in over 99% of studies, highlighting the central role of identifying tumors or abnormalities from imaging data. Screening-oriented applications also represent a substantial portion (~43.9%), reflecting the strong alignment of AI research with clinical workflows aimed at early cancer detection and population-level screening.

**Table 1:** Distribution of clinical tasks in ai-based breast cancer research (n = 608).

Clinical task	Number of papers	Percentage (%)	Avg. citations
Diagnosis	433	71.22%	24.74
Prognosis	167	27.47%	20.50
Treatment-Response	14	2.30%	3.25
Multi-task (Prog. + Treat.)	6	0.99%	23.67

While diagnostic research dominates, there has been a notable increase in prognosis-related studies, which constitute 27.47% of the corpus. These studies focus on predicting outcomes such as survival, recurrence risk, and disease progression. The growth of prognostic AI reflects an emerging trend toward precision medicine, where models aim to support long-term clinical decision-making rather than only initial detection.

In contrast, treatment-response prediction remains significantly underexplored, representing only 2.30% of studies, with an additional 0.99% addressing multi-task scenarios that combine prognosis and treatment aspects. These studies typically investigate chemotherapy response, therapy effectiveness, or treatment planning using AI models.

Overall, the evolution of AI in breast cancer research reflects a transition from task-specific diagnostic models toward broader clinical decision-support systems. While the field initially focused almost exclusively on detection and classification, recent years have seen a growing interest in prognosis and, to a lesser extent, treatment-response modeling. This trend is clearly reflected in the distribution of published studies, where diagnosis-related research dominates the field with 71.22% of papers and the highest average citation rate (24.74). Prognosis studies account for 27.47% of the literature, highlighting the increasing emphasis on personalized outcome prediction and risk assessment. In contrast, treatment-response prediction remains relatively underrepresented, comprising only 2.30% of publications and showing the lowest citation impact, which suggests that this area is still emerging and offers considerable opportunities for future research.

### 2.3 Keyword analysis and findings

The bibliometric analysis showed a varied distribution of document types among the retrieved publications. The final bibliometric dataset consisted exclusively of peer-reviewed journal articles and review papers, as illustrated in **Figure 2**. Research articles represented the dominant document type, accounting for 91.28% (555 records), while review articles comprised 8.72% (53 records). This distribution demonstrates that the field is primarily driven by original methodological and clinical research contributions, with a comparatively smaller but important body of synthesis-oriented review studies.

**Figure 2:**
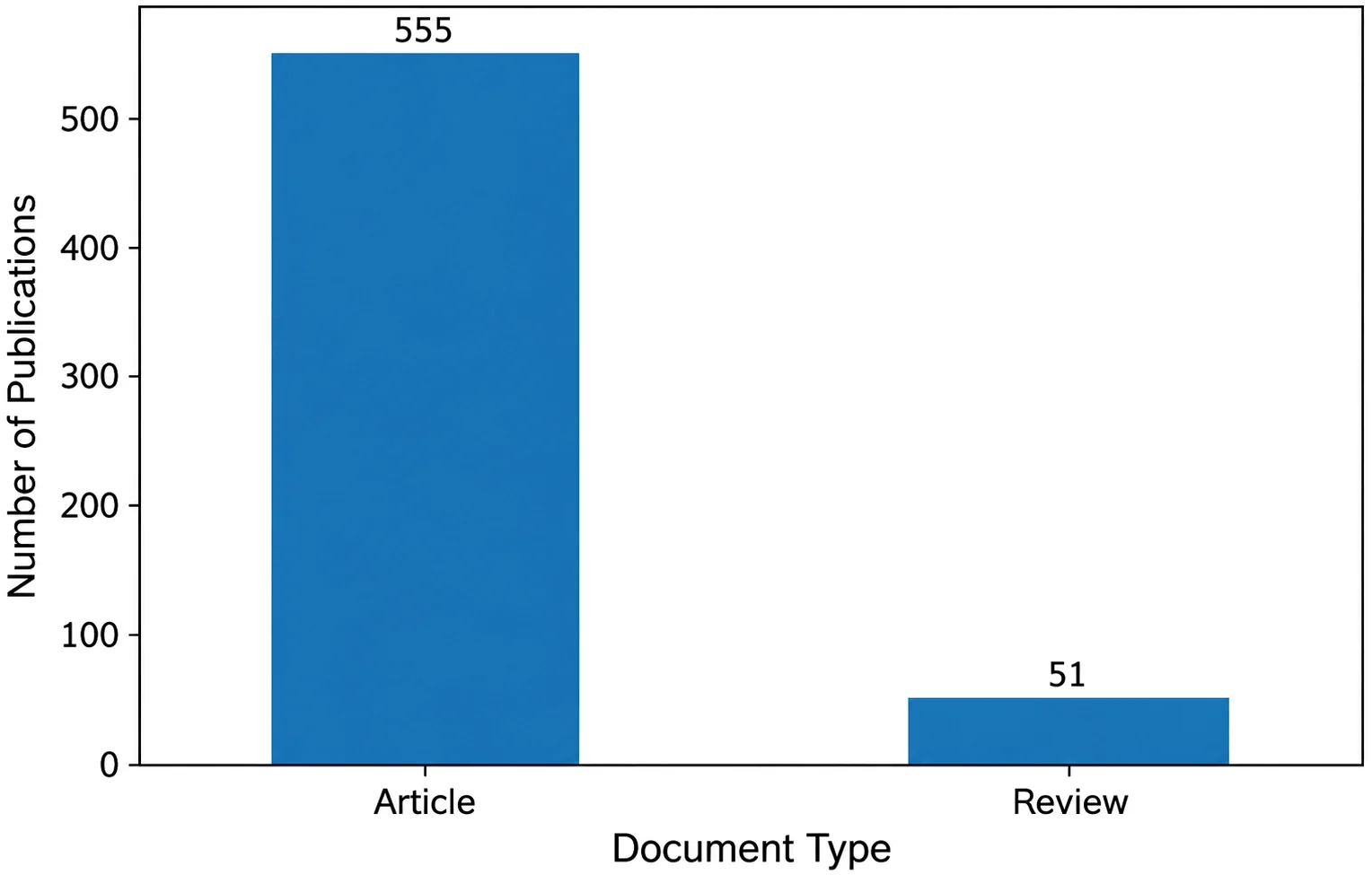
Document distribution by article type.

The annual publication trend from 2020 to 2026, shown in **Figure 3**, demonstrates a strong and continuous increase in research activity related to artificial intelligence in breast cancer imaging. Publications accounted for approximately 4% of the total literature in 2020, increasing to nearly 7% in 2021 and 15% in 2022. Research activity continued to grow substantially in 2023, contributing approximately 18% of the included studies, followed by 21% in 2024. The highest research output was observed in 2025, representing approximately 34% of the total publications included in the analysis. A noticeable decrease was observed in 2026, contributing approximately 1% of publications, which is likely attributable to incomplete indexing and partial publication coverage for the year at the time of data collection. Overall, the findings indicate a rapidly expanding research landscape, particularly after 2022, reflecting increasing scientific and clinical interest in AI-driven breast cancer detection and diagnostic technologies.

**Figure 3:**
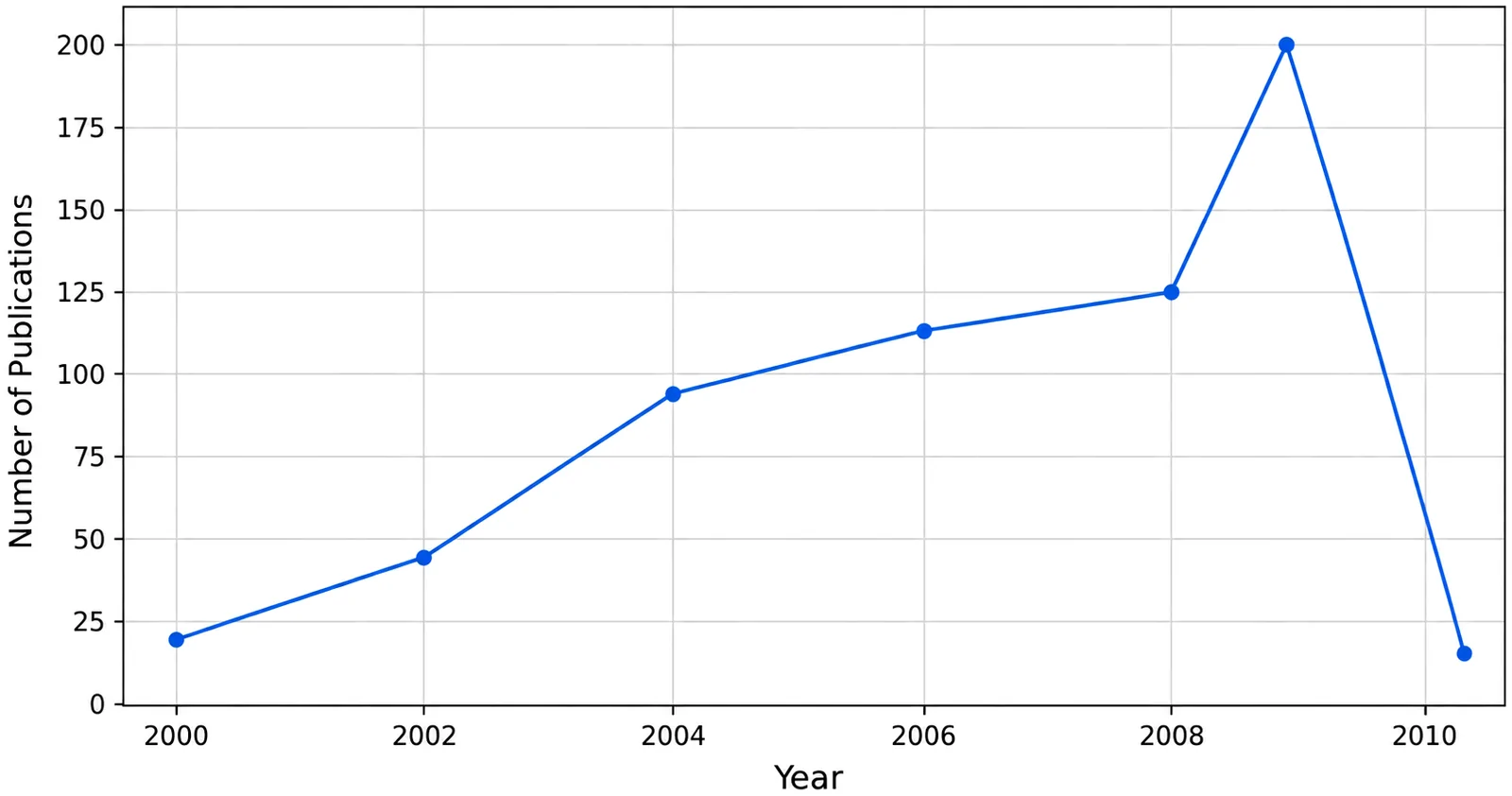
Document distribution by year.

The bibliometric analysis of the retrieved literature on breast cancer detection using deep learning indicates a robust interdisciplinary research environment. As illustrated in **Figure 4**, Computer Science accounted for the biggest percentage of articles (30.5%), indicating the essential significance of machine learning, image processing, and artificial intelligence approaches in enhancing breast cancer diagnostics. With 19.2%, engineering comes in second, with notable contributions in computational modeling, imaging hardware development, and biomedical engineering. Medicine contributes 11.9%, illustrating the therapeutic significance and expanding usage of deep learning methods in the field of medicine. Other areas, such as Mathematics (9.4%), Decision Sciences (5.6%), Physics and Astronomy (5.0%), and Biochemistry, Genetics, and Molecular Biology (3.9%), contribute smaller but substantial percentages, reflecting the multidisciplinary character of the topic.

**Figure 4:**
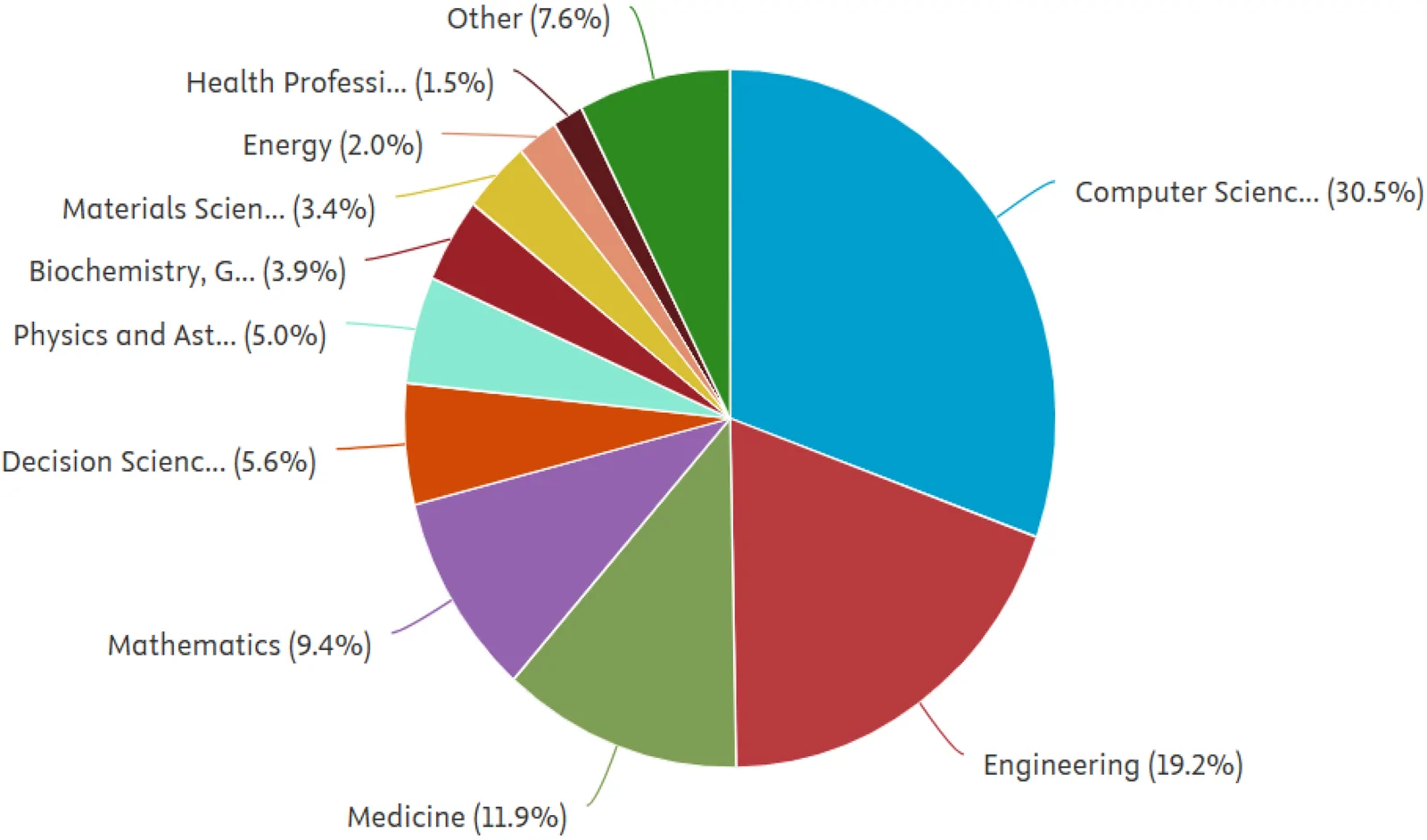
Document distribution by subject area.

**Figure 5** presents the VOSviewer keyword co-occurrence network generated from the analyzed AI-based breast cancer detection publications published between 2020 and 2026. The visualization reveals several interconnected thematic clusters representing the dominant research directions within the field. The red cluster primarily focuses on mammographic image analysis, convolutional neural networks, augmentation techniques, histopathological imaging, segmentation, and deep learning-based diagnostic frameworks. The blue cluster is strongly associated with artificial intelligence systems, sensitivity analysis, assessment methods, breast imaging reporting, and clinical evaluation metrics, reflecting the increasing integration of AI into clinical diagnostic workflows. Meanwhile, the green cluster emphasizes implementation strategies, interpretability, Grad-CAM visualization, benefits of AI-assisted diagnosis, and model explainability. The yellow cluster mainly represents machine learning algorithms, prediction models, support vector machines, logistic regression, optimization methods, and traditional classification approaches.

**Figure 5:**
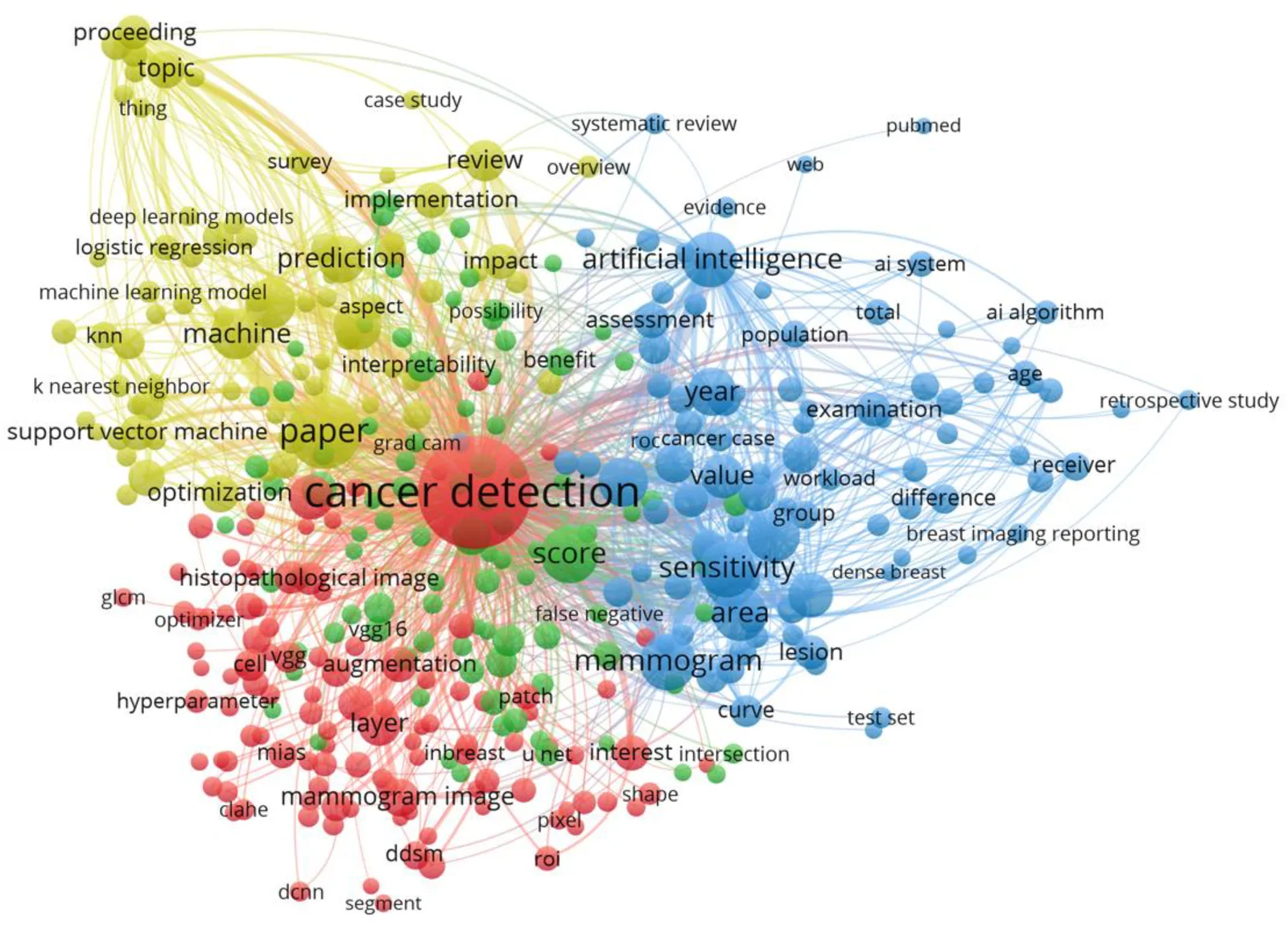
A keyword co-occurrence network.

In the generated network, node size represents the frequency of keyword occurrence, whereas the connecting links indicate co-occurrence relationships between keywords. Stronger and denser links reflect closer thematic associations among research topics. The central positioning of terms such as “cancer detection, “ “artificial intelligence, “ “mammogram, “ and “machine learning” demonstrates their dominant role within the intellectual structure of the field. Furthermore, the overlap among clusters indicates the interdisciplinary nature of AI-driven breast cancer detection research, combining medical imaging, computer vision, machine learning, explainable AI, and clinical decision-support systems.

The keyword co-occurrence network was generated using VOSviewer software based on association-strength normalization. Keywords meeting the predefined minimum occurrence threshold were included in the final visualization, and clusters were automatically generated according to keyword co-occurrence relationships within the dataset.

### 2.4 Advanced bibliometric analysis of AI-based breast cancer detection research

To provide a deeper understanding of the structure, impact, and evolution of research in AI-based breast cancer detection, an advanced bibliometric analysis was conducted using the curated corpus. Beyond basic descriptive statistics, this section applies established bibliometric laws and analytical techniques to uncover influential contributions, research dynamics, and emerging trends within the field.

#### 2.4.1 Most cited papers

The most highly cited studies in breast cancer AI research are predominantly focused on detection and diagnosis using mammography, digital breast tomosynthesis, and deep learning techniques. **Table 2** shows that convolutional neural networks (CNNs), hybrid deep learning models, and AI-assisted radiologist assessment are central themes in influential publications. Most of the top-cited papers were published between 2020 and 2021, reflecting the rapid growth of AI applications in medical imaging during this period. The dominance of imaging-based diagnostic studies among the most cited papers further confirms that diagnosis remains the most mature and impactful area in breast cancer AI research.

**Table 2:** Top 10 most cited papers derived from the 608 paper corpus.

Rank	Title	Authors	Year	Citations
1	An anatomization on breast cancer detection and diagnosis employing multi-layer perceptron neural network (MLP) and convolutional neural network (CNN)	Desai, M.; Shah, M.	2021	381
2	Changes in cancer detection and false-positive recall in mammography using artificial intelligence: a retrospective, multireader study	Kim, H.-E.; Kim, H.H.; Han, B.-K.; Kim, K.H.; et al.	2020	380
3	Evaluation of Combined Artificial Intelligence and Radiologist Assessment to Interpret Screening Mammograms	Schaffter, T.; Buist, D.S.M.; Lee, C.I.; Nikulin, Y.; et al.	2020	326
4	Improved Breast Cancer Classification Through Combining Graph Convolutional Network and Convolutional Neural Network	Zhang, Y.-D.; Satapathy, S.C.; Guttery, D.S.; et al.	2021	306
5	Robust breast cancer detection in mammography and digital breast tomosynthesis using an annotation-efficient deep learning approach	Lotter, W.; Diab, A.R.; Haslam, B.; Kim, J.G.; et al.	2021	296
6	Artificial intelligence for breast cancer detection in mammography and digital breast tomosynthesis: state of the art	Sechopoulos, I.; Teuwen, J.; Mann, R.	2021	218
7	Breast cancer detection by leveraging Machine Learning	Anji Reddy, A.R.; Soni, B.; Reddy, S.R.	2020	203
8	Stochastic Dilated Residual Ghost Model for Breast Cancer Classification	Kashyap, R.	2023	193
9	Artificial intelligence for breast cancer detection in screening mammography in comparison to double reading by radiologists	Dembrower, K.; Crippa, A.; Colón, E.; Eklund, M.; et al.	2023	183
10	Applying deep learning in digital breast tomosynthesis for automatic breast cancer detection: a review	Bai, J.; Posner, R.; Wang, T.; Yang, C.; Nabavizadeh, S.A.; et al.	2021	141

**Figure 6** highlights the most productive journals publishing research on AI applications in breast cancer analysis. Biomedical Signal Processing and Control ranks first with the highest number of publications, indicating its strong focus on medical imaging and AI-driven healthcare research. IEEE Access and Scientific Reports follow closely, reflecting the interdisciplinary nature of breast cancer AI studies across engineering, computer science, and medical domains. Journals such as Diagnostics, Computers in Biology and Medicine, and Radiology: Artificial Intelligence further demonstrate the growing integration of artificial intelligence into clinical imaging and diagnostic workflows. Overall, the distribution of publications across both medical and engineering journals emphasizes the highly multidisciplinary character of modern breast cancer research and the increasing collaboration between healthcare and AI communities.

**Figure 6:**
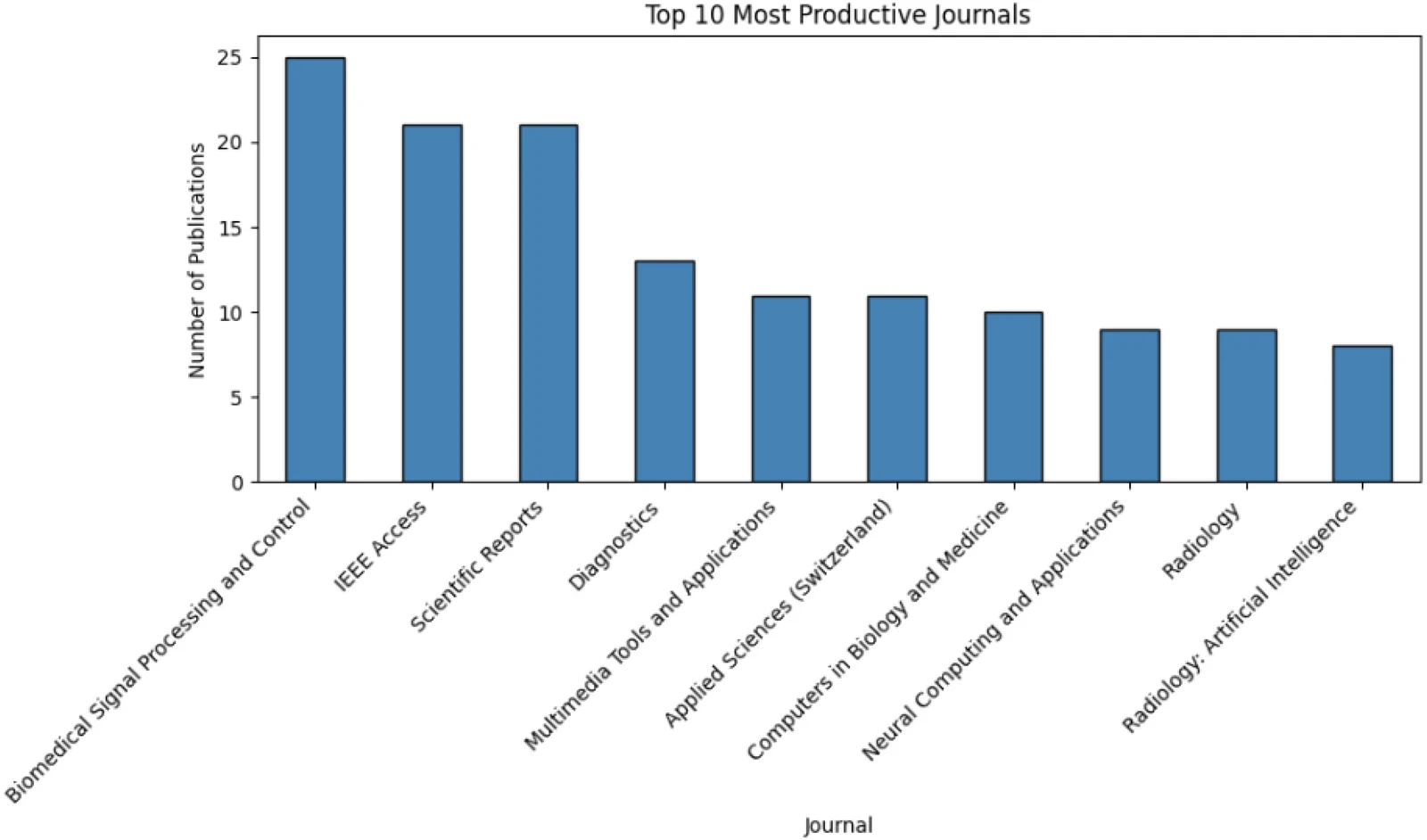
The top 10 most productive journals.

#### 2.4.2 Citation growth trend analysis

The citation growth analysis illustrated in **Figure 7** revealed a substantial increase in research influence starting in 2020, coinciding with the widespread adoption of deep learning architectures for breast cancer detection and classification. Total citation counts increased from 2, 059 citations in 2020 to 2, 681 citations in 2021, while maintaining consistently high citation activity during 2022 and 2023. In recent years, such as 2025 and 2026, exhibited lower citation counts; this reduction is primarily attributed to the limited time available for newly published articles to accumulate citations. Overall, the results confirm the accelerating scientific interest and rapid development of artificial intelligence methodologies in breast cancer imaging and diagnosis between 2020 and 2026.

**Figure 7:**
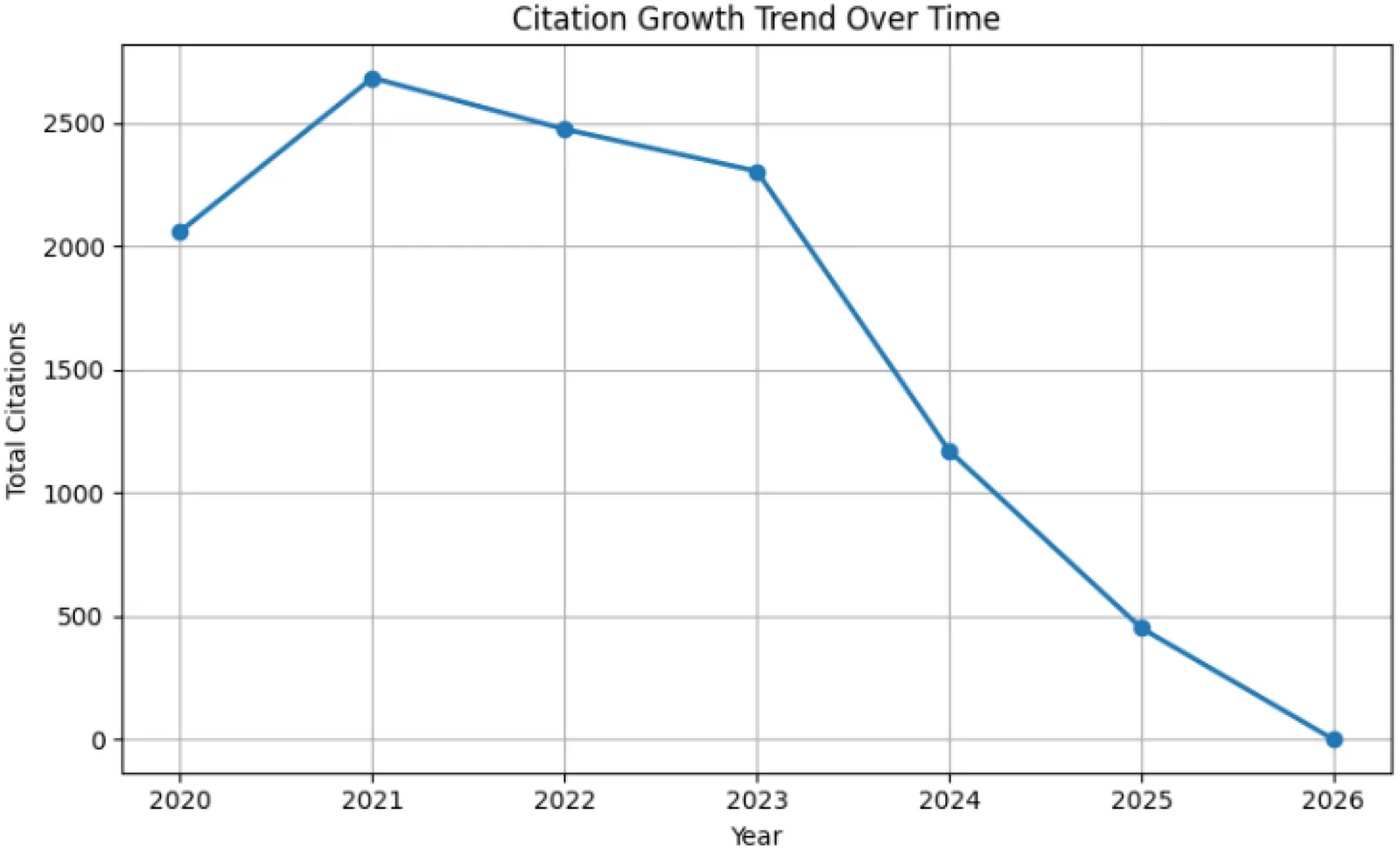
Number of citations per year.

#### 2.4.3 Journal productivity and Bradford’s law analysis

Bradford’s Law is a bibliometric principle that describes the distribution of scientific publications across journals, stating that a relatively small core set of journals accounts for a large proportion of publications in a given field, while the remaining articles are scattered across an increasing number of less productive journals. In line with Bradford’s Law, the journal-level analysis revealed that research output in AI-based breast cancer detection between 2020 and 2026 was concentrated within a relatively small group of highly productive journals. **Figure 8** describes that *Biomedical Signal Processing and Control* ranked as the most productive journal with 25 publications, followed by *IEEE Access* and *Scientific Reports*, each contributing 21 publications. Additional influential journals included *Diagnostics*, *Computers in Biology and Medicine*, and *Radiology: Artificial Intelligence*, highlighting the interdisciplinary nature of the field spanning biomedical engineering, medical imaging, artificial intelligence, and clinical radiology. Moreover, Bradford’s law-inspired analysis demonstrated that a limited core set of journals accounted for a substantial proportion of the published literature, indicating the emergence of specialized publication venues that dominate AI-driven breast cancer research dissemination.

**Figure 8:**
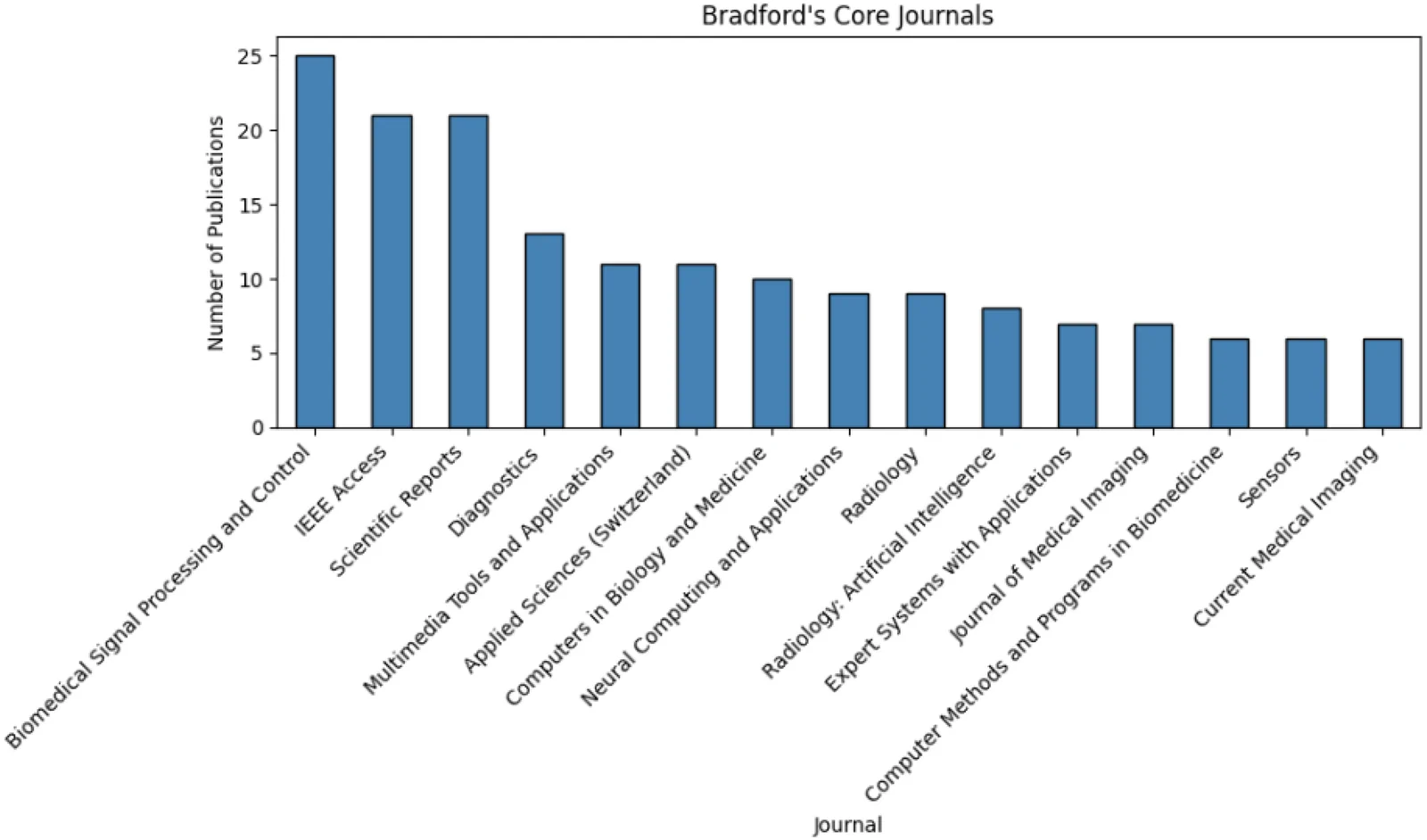
Journal-level analysis with the number of publications.

#### 2.4.4 Author productivity and Lotka’s law analysis

Lotka’s Law is a bibliometric principle that describes the distribution of scientific productivity among authors, stating that the number of authors publishing n papers is approximately proportional to 1/n² of those publishing a single paper, meaning that a small number of authors contribute a large share of publications while most authors publish only one or a few papers. In line with Lotka’s Law, an author productivity analysis was performed to evaluate contribution patterns among researchers in the field between 2020 and 2026. The findings demonstrated a highly skewed productivity distribution, where most authors contributed only a single publication, while a significantly smaller group of researchers produced multiple publications. As shown in **Figure 9**, More than 2, 700 authors were associated with only one publication, whereas only a limited number of authors contributed to more than ten publications. This productivity pattern is consistent with Lotka’s law and reflects the collaborative and rapidly evolving nature of artificial intelligence research in breast cancer detection, where a concentrated group of highly active researchers drives much of the scientific advancement.

**Figure 9:**
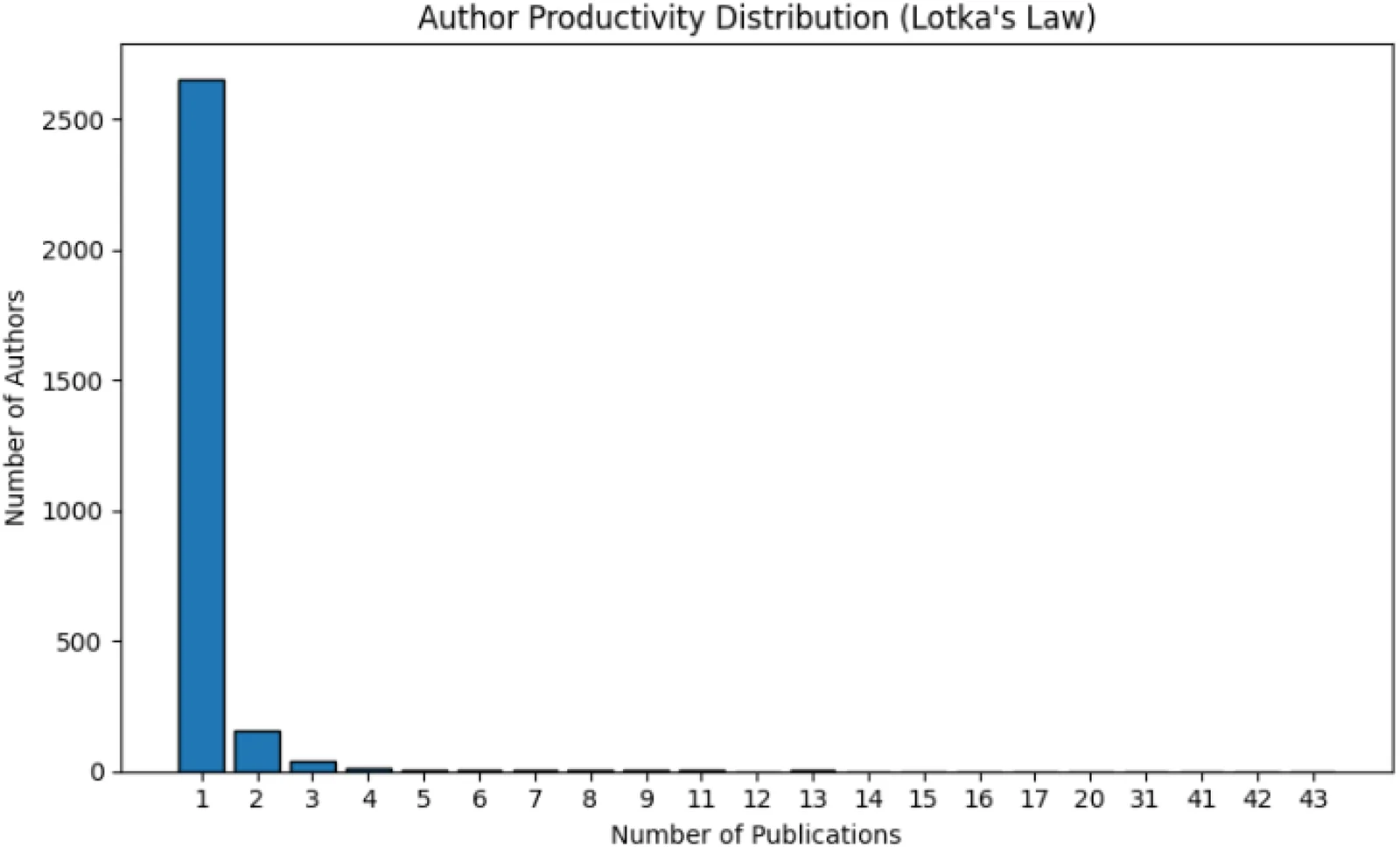
Author productivity with the number of publications.

#### 2.4.5 Temporal evolution of research themes

Temporal keyword analysis revealed a clear evolution of research themes between 2020 and 2026 is described in **Figure 10**. Early studies conducted in 2020 and 2021 primarily focused on deep learning, convolutional neural networks, mammography, and machine learning approaches for breast cancer diagnosis. Beginning in 2022, the field experienced substantial growth in artificial intelligence-driven diagnostic systems, with keywords such as “breast cancer detection, “ “deep learning, “ “artificial intelligence, “ and “machine learning” becoming increasingly dominant. Between 2023 and 2025, research activity intensified around mammography-based AI systems, explainable artificial intelligence, and advanced imaging techniques. Furthermore, recent keywords appearing in 2026, including “digital breast tomosynthesis” and “saliency maps, “ indicate a growing emphasis on interpretable AI frameworks and clinically deployable diagnostic solutions.

**Figure 10:**
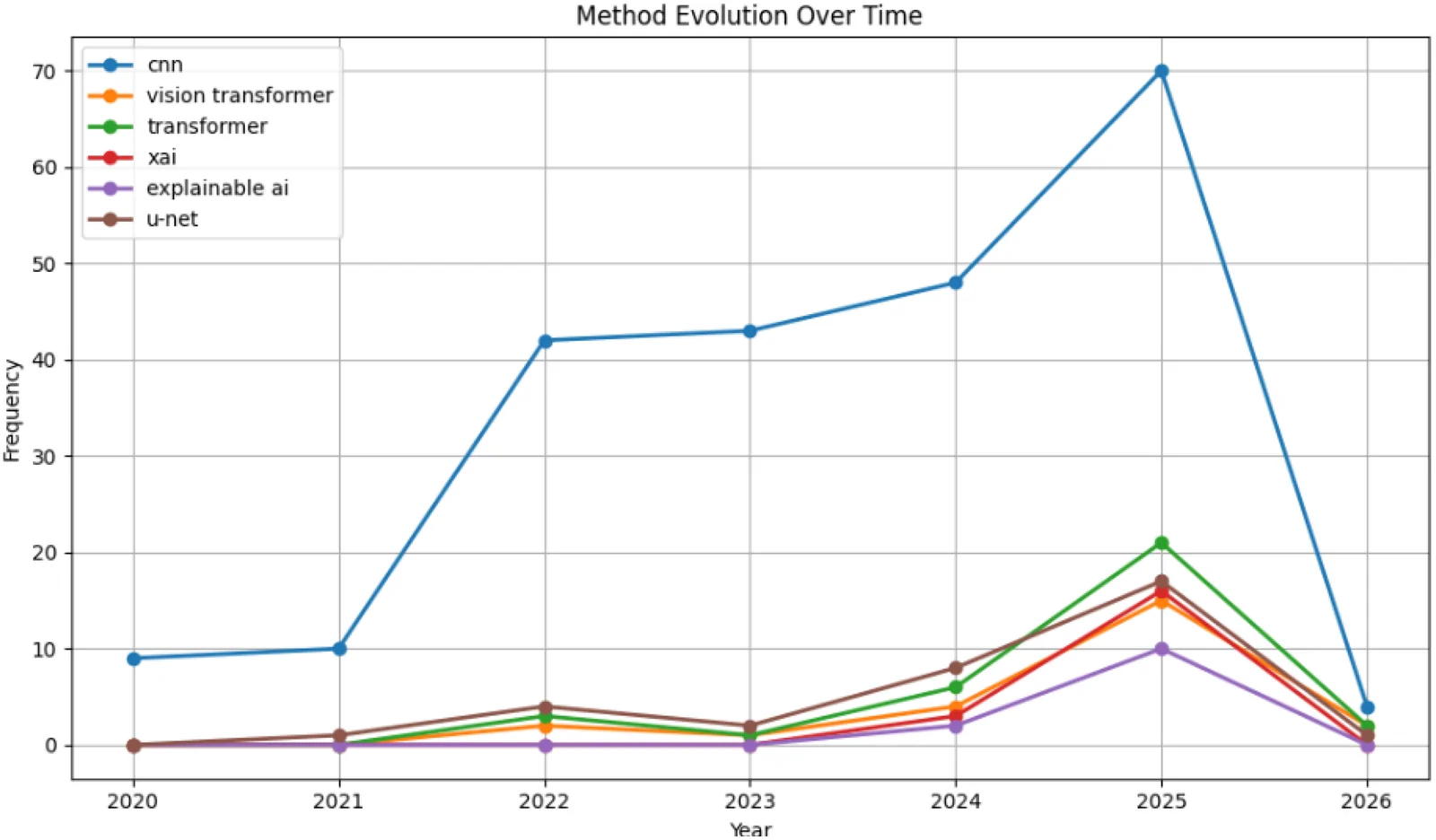
Temporal evolution of dominant research keywords in AI-based breast cancer detection from 2020 to 2026.

The bibliometric analysis provides a comprehensive understanding of the intellectual structure and developmental trajectory of AI-based breast cancer detection research between 2020 and 2026. Beyond conventional publication counts, the study incorporates Citation Impact Analysis, Citation Growth Trend Analysis, Journal Productivity and Bradford’s Law Analysis, Author Productivity and Lotka’s Law Analysis, and Temporal Evolution of Research Themes. The findings collectively demonstrate that the field has evolved rapidly during recent years, driven primarily by advances in deep learning, convolutional neural networks, transformer-based architectures, explainable artificial intelligence, and mammographic image analysis. Furthermore, the integration of citation metrics, temporal keyword evolution, Bradford’s law-inspired journal evaluation, and Lotka’s law-inspired author productivity assessment provides deeper insights into influential studies, dominant publication venues, research productivity patterns, and emerging methodological trends shaping the future of AI-assisted breast cancer diagnosis. Bradford’s law and Lotka’s law were applied descriptively to analyze journal and author productivity distributions.

#### 2.4.6 Burst map and keyword growth intensity

The quantitative hotspot analysis in **Table 3** reveals a clear transition in AI-based breast cancer research. While deep learning and CNNs remain the dominant foundational technologies, their relatively lower burst ratios indicate a mature and saturated research area. In contrast, emerging topics such as Explainable AI (burst = 1.00), Transformers/ViT (~0.89), and multimodal learning (~0.92) exhibit strong growth signals, identifying them as the current and future hotspots of the field. This shift reflects a broader paradigm transition from accuracy-focused models toward interpretable, multimodal, and clinically robust AI systems.

**Table 3:** Quantitative hotspots analysis (keyword frequency, trends, and burst).

Keyword	Total frequency	Share (%)	Recent count (2023–2026)	Recent share (%)	Burst ratio	Trend classification
Deep learning	387	63.7%	281	46.2%	0.73	Mature/Saturated
CNN	106	17.4%	81	13.3%	0.76	Stable Core
Transfer learning	85	14.0%	64	10.5%	0.75	Stable Core
Attention	33	5.4%	31	5.1%	0.94	Rapid Growth
Transformer	27	4.4%	24	3.9%	0.89	Fast Adoption
Vision Transformer (ViT)	19	3.1%	17	2.8%	0.89	Rapid Growth
Explainable AI (XAI)	35	5.8%	22	3.6%	**1.00**	Emerging Hotspot
GAN	17	2.8%	13	2.1%	0.76	Moderate Growth
Multimodal	13	2.1%	12	2.0%	0.92	Emerging Trend

Bold indicates Emerging Hotspot.

The burst map in **Figure 11** shows a transition from mature deep learning and CNN-based approaches toward emerging paradigms, particularly explainable AI, transformer architectures, and multimodal learning. These trends highlight a shift in research priorities from pure performance improvement toward interpretability, integration of diverse data sources, and clinically applicable AI systems.

**Figure 11:**
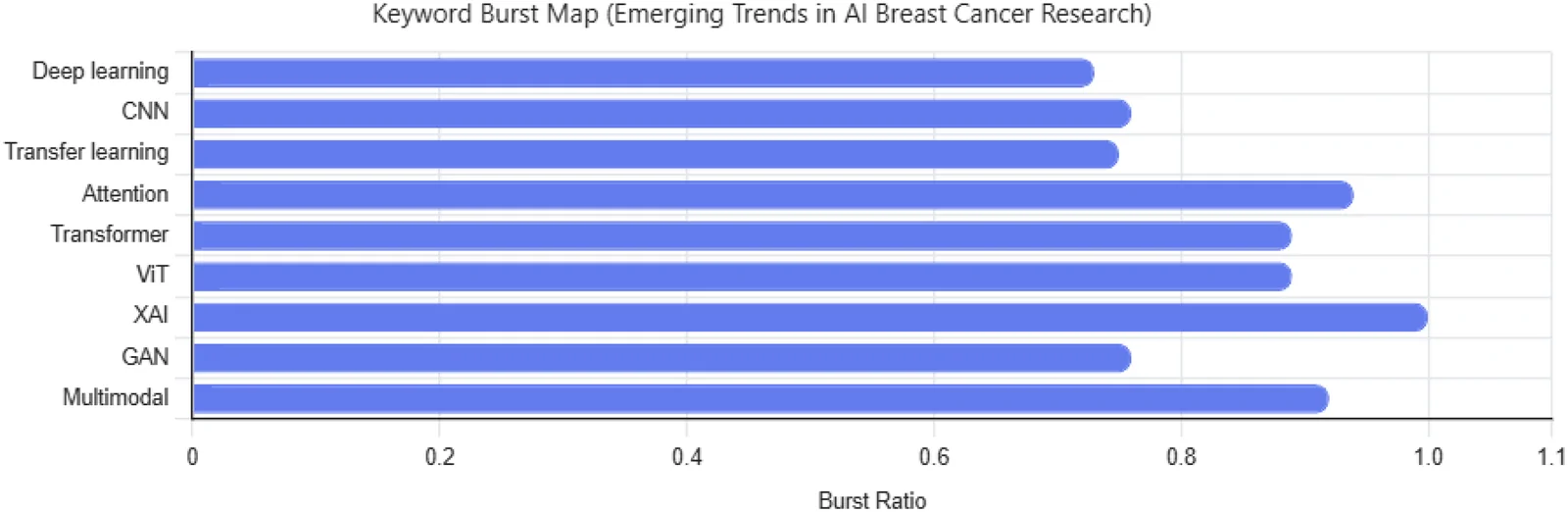
Burst map visualization based on keyword growth intensity.

Building on the bibliometric findings, which reveal rapid growth alongside increasing methodological diversity in AI-driven breast cancer research, a focused thematic examination is necessary to contextualize these patterns and derive actionable insights. The analysis highlights substantial heterogeneity across imaging modalities, data resources, algorithmic practices, and evaluation strategies, as well as a growing emphasis on clinical trust and reproducibility. Accordingly, the following sections examine key themes emerging from the bibliometric evidence, including imaging modalities, datasets and software ecosystems, reproducibility considerations, explainable AI techniques, and hyperparameter optimization strategies. These thematic analyses provide the foundation for a final discussion that synthesizes current challenges and outlines future directions toward reproducible, interpretable, and clinically integrated AI systems for breast cancer care.

## 3 Imaging modalities

Imaging modalities are critical in various stages of breast cancer care continuum, with their clinical applicability differing by disease stage, patient characteristics, and diagnostic goals. Mammography is the mainstay of population-based breast cancer screening, and the only modality that has proven to lower breast cancer mortality in average risk women by facilitating early detection of asymptomatic disease (5, 6). While mammography is far from perfect, and has a few known drawbacks, such as false negatives and interval cancers, the benefits of early detection, widespread availability, low cost, and a uniform system to categorize mammographic findings (e.g., BI-RADS) are noteworthy (7). The less sensitive a modality is, the more likely a woman is to suffer from one of these problems. The use of adjunctive modalities has, therefore, become a norm when screening mammography is employed. For evaluating mammographically detected lesions, adjunctive breast ultrasound is one such tool that is particularly good for screening dense breasts. It does not use ionizing radiation, is less expensive, and is better, as a first line modality, for screening, especially when looking for solid versus fluid-filled (cystic) masses but is more prone to false positive results (8). The breast imaging modality with the highest sensitivity is Magnetic Resonance Imaging (MRI). MRI is especially important in high-risk screening, pre-operative staging, and assessing post-neoadjuvant therapy response.

Its soft tissue contrast and functional imaging capabilities are unrivaled; however, these are outweighed by high costs, low availability, reduced specificity, and the risk of overdiagnosis (9). More sophisticated techniques like PET/CT and contrast-enhanced imaging are almost always reserved for the staging and monitoring of metastatic disease, as they provide more radiation and are less sensitive for small primary tumors (10). As such, these modalities are neither interchangeable nor expendable; knowing as such requires the evaluator to balance diagnostic accuracy, patient risk, and resource availability against the particular step in the breast cancer care continuum.

### 3.1 Distribution of clinical tasks across imaging modalities

The analysis of the dataset (n = 608) reveals that mammography is the dominant imaging modality, appearing in 256 studies (42.11%), which is substantially higher than any other modality. In comparison, ultrasound is used in 100 studies (16.45%), making mammography approximately 2.5 times more prevalent than ultrasound-based approaches. Histopathology-based studies account for 87 papers (14.31%), reflecting its importance in detailed tissue-level diagnosis, while MRI is relatively less frequent, appearing in only 21 studies (3.45%). The remaining modalities—including thermography, digital breast tomosynthesis (DBT), and multimodal approaches, are more dispersed and collectively account for roughly 24% of the dataset.

Distribution of Clinical Tasks Across Imaging Modalities, as shown in **Table 4** highlights a strong dependency between imaging type and clinical purpose. Mammography is predominantly used for diagnostic tasks, especially screening and detection, reflecting its central role in clinical practice. In contrast, histopathology shows a comparatively higher association with prognosis, due to its ability to provide detailed cellular-level information for outcome prediction. Ultrasound demonstrates a more balanced distribution across diagnosis and prognosis, while MRI remains limited but specialized. Notably, treatment-response prediction is minimally explored across all modalities, underscoring a significant gap and opportunity for future AI research.

**Table 4:** Distribution of clinical tasks across imaging modalities.

Imaging modality	Diagnosis	Prognosis	Treatment
Mammography	181	87	3
Ultrasound	67	31	3
Histopathology	58	31	0
MRI	19	4	0

### 3.2 Mammography

Mammography remains the primary modality for AI-assisted breast cancer screening and diagnosis due to the availability of extensive screening cohorts and public datasets. Typical tasks are cancer detection, triage/reading assistance, segmentation of suspicious findings, and classification in the BI-RADS system. The included corpus has real-world implementation work in population-based mammography screening (11) and algorithmic work in BI-RADS classification using vision transformers (12) and mass segmentation using hybrid transformer–UNet designs (13).

### 3.3 Ultrasound

Most breast cancer AI studies that utilize ultrasound focus on lesion detection and classification as benign or malignant, as there can be a large variation in operator performance, as well as variable acquisition of the ultrasound sequences. Ultrasound is studied as a stand-alone modality, but also as a component of multimodal systems. For example, multimodal systems incorporating ultrasound are described in (14).

### 3.4 Magnetic resonance imaging.

Within the corpus, the utilized studies on MRI are aimed at higher-level studies, like multiparametric acquisitions lesions detection, segmentation, and characterization. Some studies also focus on decentralized and privacy-preserving areas of study. For instance, there are studies on AI for MRI screening (15) and decentralized (swarm) learning for breast MRI (16) analyzing breast MRI lesion detection as object detection (17).

### 3.5 Histopathology and digital pathology

AI in digital pathology studies analyzes tissue and cellular morphology for supporting the classification of the tumor and the prognosis. The 2024–2025 corpus indicates the ongoing shift from standard CNN architectures to those incorporating attention and transformer-style components. Neural networks as explainable systems for detection of breast cancer are discussed in (18).

### 3.6 Multimodal integration

Numerous studies in 2024–2025 focus on the development of multimodal systems that combine multiple data streams (e.g. various imaging modalities, and/or other non-imaging inputs). The corpus also includes multimodal transformer-based diagnostic (14, 19).

## 4 Datasets, software, and reproducibility

The reviewed studies rely on a combination of widely used public datasets and newly developed institutional datasets. Mammography public datasets like DDSM, CBIS-DDSM, INbreast, and MIAS are often used across literature, allowing for comparisons across the literature and for lesion detection, segmentation, and classification. Moreover, datasets continue to emerge, including a forthcoming 2025 mammographic dataset which aims to enhance the ability to detect and classify breast cancer (20).

### 4.1 Dataset ecosystems and benchmarking

The evaluated works utilize both mainstream, public datasets and fresh, proprietary institutional datasets. Public narratives in the corpus highlight the reliance on mainstream datasets in breast cancer research, such as DDSM, CBIS-DDSM, INbreast, and MIAS, as they are routinely referenced in the corpus and promote benchmarking for the evaluation of lesion detection, segmentation, and classification. New dataset contributions are also present, for instance, the corpus includes a 2025 dataset release aimed at improving mammographic breast cancer detection and classification (20).

#### 4.1.1 Datasets and analytical software

Integration of clinical, genomic, imaging, and epidemiological data is part of the breast cancer machine learning studies (datasets). In the case of tumor classification, the Wisconsin Breast Cancer Dataset is often cited (21). For the studies TCGA-BRCA and METABRIC, there is reference to molecular characterization studies, that merge clinical and molecular datasets related to cancer (22, 23). As for the studies’ foundation, SEER stands for Surveillance, Epidemiology, and End Results and describes the cancer’s onset, treatment, and survival across different populations (24). These datasets focus on mammography studies aimed at the design and development of radiomic and deep learning models (25). GEO (Gene Expression Omnibus) is the most prominent breast cancer dataset, allowing for inter-study validation and meta-analysis (26) (**Table 5**). In (27) data is analyzed, while in Python the machine learning and imaging data are processed (28), and in SAS or Stata the population datasets are processed.

**Table 5:** Publicly available breast cancer datasets, analytical software, URL access and usage in the corpus.

Dataset	Data source	~ Sample size	Key features	Data type	Analytical software	Dataset URL	Usage in the corpus
Wisconsin Breast Cancer Dataset (WBCD)	UCI Machine Learning Repository	~569 patients	Tumor morphology, texture, symmetry, smoothness, compactness	Clinical/Imaging-derived	R, Python (scikit-learn), MATLAB	https://archive.ics.uci.edu/ml/datasets/Breast+Cancer+Wisconsin+(Diagnostic)	19 papers
TCGA-BRCA	The Cancer Genome Atlas	~1, 100 patients	Somatic mutations, RNA sequencing, DNA methylation, copy number variation, clinical outcomes	Genomic + Clinical	R (Bioconductor), Python	https://portal.gdc.cancer.gov/projects/TCGA-BRCA	0
METABRIC	METABRIC Consortium	~2, 000 patients	Gene expression, copy number alterations, intrinsic molecular subtypes, survival outcomes	Genomic + Clinical	R, Python	https://ega-archive.org/studies/EGAS00000000083	0
SEER Breast Cancer Registry	National Cancer Institute (SEER)	>1, 000, 000 cases	Demographics, tumor stage, grade, treatment modalities, survival	Epidemiologic/Clinical	SAS, Stata, R	https://seer.cancer.gov	1 papers
CBIS-DDSM	The Cancer Imaging Archive (TCIA)	~3, 000 mammograms	Breast mass morphology, calcifications, pathology-confirmed labels	Imaging (Radiomics)	Python, MATLAB	https://www.cancerimagingarchive.net/collection/cbis-ddsm/	32 papers
Gene Expression Omnibus (GEO)	NCBI	Dataset-specific	Microarray and RNA-seq gene expression profiles	Genomic	R (GEOquery), Python	https://www.ncbi.nlm.nih.gov/geo/	18 papers
UK Biobank (Breast Cancer Subset)	UK Biobank	~500, 000 participants	Genetic variants, imaging, lifestyle factors, clinical diagnoses	Multi-modal	R, Python	https://www.ukbiobank.ac.uk/	0

A total of 168 explicit dataset mentions were identified across the reviewed studies. Among these, MIAS and BreakHis emerge as the most frequently used datasets, each appearing 37 times (22.02%), highlighting their widespread adoption for both mammography and histopathology analysis. INbreast follows closely with 33 mentions (19.64%), and CBIS-DDSM appears in 32 studies (19.05%), confirming its strong presence as a key benchmark dataset for mammography-based research. Meanwhile, BUSI (ultrasound dataset) is referenced in 29 studies (17.26%), reflecting growing interest in ultrasound imaging, though still slightly less dominant compared to mammography datasets.

Overall, mammography datasets (MIAS, INbreast, CBIS-DDSM combined) account for approximately 60.7% of the top dataset mentions, reinforcing the central role of mammographic imaging in breast cancer AI research. In contrast, ultrasound (BUSI) and histopathology (BreakHis) datasets collectively contribute around 39.3%, indicating an increasing but still secondary focus on complementary imaging modalities.

#### 4.1.2 Access to referenced datasets

The datasets discussed in this section are publicly accessible through their respective repositories. The Breast Cancer Wisconsin dataset is available at the UCI Machine Learning Repository. You can access the TCGA-BRCA data through the Genomic Data Commons portal. The METABRIC data is available at the European Genome-Phenome Archive. SEER data can be obtained through a request to the National Cancer Institute. The imaging data for the CBIS-DDSM is accessible through The Cancer Imaging Archive. The gene expression data is housed in the NCBI Gene Expression Omnibus. UK Biobank data (29) is available to approved researchers through a formal application process.

### 4.2 Software frameworks and tools

The most widely reported tools and frameworks are deep learning software such as Keras and PyTorch. For instance, Keras is noted as being employed as a vision transformer for mammography (12) and PyTorch is noted as being employed in attention-based classification (30). Tools selection affects reproducibility in terms of their dependency management, versioning, and how easily training scripts and inference code can be shared. **Table 6** describes integrated systems of software and data and their relevant applications in bioinformatics and medical imaging.

**Table 6:** Software frameworks and tools for breast cancer data analysis.

Software/framework	Open source?	Primary use/important features	URL	Reference (APA)
TensorFlow	Yes	Deep learning library for building and training neural networks; widely used for medical image classification and deep models in cancer research.	https://www.tensorflow.org/	(Google Brain Team, 2015/ongoing) (31)
PyTorch	Yes	Deep learning framework optimized for dynamic computational graphs; popular for CNNs, transfer learning, and research models.	https://pytorch.org	(Paszke et al., 2019) (32)
MONAI (Medical Open Network for AI)	Yes	Domain-optimized deep learning for medical imaging; provides image segmentation, classification, and registration tools tailored to biomedical applications.	https://project-monai.github.io/	(MONAI Community/NVIDIA & NIH) (33)
Bioconductor	Yes	R-based bioinformatics platform for genomic data analysis (differential expression, sequencing, visualization).	https://www.bioconductor.org/	(Bioconductor Project, 2025) (34)
Galaxy	Yes	Web-based workflow platform for reproducible genomic and bioinformatics analyses without programming; integrates many tools in a user interface.	https://galaxyproject.org	(Afgan et al., Galaxy Project) (35)
scikit-learn	Yes	Python library for traditional ML (SVM, random forest, clustering); used in breast cancer classification & feature selection.	https://scikit-learn.org/	(Pedregosa et al., 2011) (28)
mlpack	Yes	High-performance C++ ML library with many classical algorithms (classification, regression, clustering).	https://www.mlpack.org/	(mlpack community, 2025) (36)
Dask	Yes	Parallel computing library for Python; scales ML workflows and integrates with scikit-learn.	https://dask.org/	(Dask community, 2025) (37)

MONAI Bioconductor is an example of software ecosystems tailored to a specific domain. It is the MONAI toolkit, which streamlines domain-specific medical AI to facilitate rapid imaging segmentation, classification, and volumetric research. Bioconductor is an example of a software ecosystem for a specific domain. It is a collection of R-based genomic and transcriptomic packages that serve omics studies through differential expression, sequencing alignment, and other related functions. Galaxy is a platform that allows users to conduct reproducible genomic analyses without the need for advanced programming skills. Dask, mlpack, scikit-learn are fundamental, highly scalable, and commercially available toolkits for traditional machine learning and the advanced computing of cancer datasets. SAS, Stata (38), MATLAB are commercially available advanced software applications used for image analysis, modeling, and epidemiological studies, commonly in clinical research, and are used for advanced image analysis, modeling, and epidemiological studies.

### 4.3 Clinical tasks and typical evaluation metrics

The classification of studies by clinical task is critical because it aligns AI research with real clinical requirements. Diagnostic models address immediate needs in cancer detection and screening, making them highly prevalent and clinically impactful. In contrast, prognostic and treatment-related models support long-term decision-making and personalized medicine, which are increasingly important but require more complex data and modeling strategies. Understanding this distribution helps identify imbalances in research focus, such as the overrepresentation of diagnostic studies and the underdevelopment of treatment-response modeling.

AI-based breast cancer research demonstrates a clear gradient in performance and maturity across clinical tasks as shown in **Table 7**. Diagnostic models achieve the highest accuracy and AUC due to well-defined imaging datasets and standardized evaluation protocols, while prognostic models show moderate predictive performance using survival-based metrics such as the C-index. In contrast, treatment-response prediction remains underdeveloped, with limited studies reporting modest AUC values and inconsistent pCR prediction results, highlighting a critical gap in the literature.

**Table 7:** Clinical tasks and typical evaluation metrics.

Clinical task	Common metrics	Typical performance range (from corpus)	Example outcomes
Diagnosis	Accuracy, AUC, Sensitivity, Specificity, F1-score	Accuracy: 90–99% AUC: 0.85–0.99	• CNN/Transformer models often achieve >95% accuracy • Some studies report AUC up to 0.99
Prognosis	C-index, Hazard Ratio (HR), AUC, Survival curves	C-index: 0.65–0.90 AUC: 0.70–0.90	• Deep models predict survival with C-index ~0.75–0.85 • Risk stratification models show moderate performance
Treatment-response	AUC, Accuracy, pCR (pathological complete response), ROC	AUC: 0.65–0.85 pCR prediction: moderate	• Limited studies, but typically lower performance than diagnosis • pCR prediction remains inconsistent

## 5 Advances in transformer-based and hybrid deep learning models for breast cancer diagnosis

Artificial intelligence, deep learning, prediction, and mammography were found to be the most popular research subjects in a bibliometric study, and recent term evolution suggested that transformer-based architecture was gaining popularity. The emergence of Vision Transformers (ViTs) and hybrid CNN–Transformer techniques, indicates a shift toward architectures capable of modeling long-range dependencies in images, even as convolutional neural networks remain the most widely studied models. A special section on Transformer-based techniques for breast cancer imaging was added in response to this new trend.

Transformer-based architectures, especially the Vision Transformer (ViT), have recently become prominent in medical image analysis. They excel at modeling long-range dependencies and global context through self-attention mechanisms, effectively overcoming some limitations of traditional convolutional neural networks (CNNs) (39). Unlike CNNs, which mainly focus on localized receptive fields and hierarchical features, ViTs process images as sequences of patch embeddings and use multi-head self-attention to capture comprehensive feature interactions across the entire image. This approach is particularly useful for breast cancer diagnosis, where lesions often show subtle, diverse, and spread-out patterns that convolutional methods might miss. Recent research shows transformer-based models, including ViT and Swin Transformer variants, perform as well as or better than advanced CNNs in breast cancer classification, mass detection, and malignancy prediction on mammography, ultrasound, and MRI images (40, 41). Additionally, hybrid Transformer models enhance diagnostic accuracy by combining the proficiency of CNNs in local feature extraction with the ability of Transformers to analyse global context. This integration results in more robust, generalizable, and interpretable clinical decision-support systems (42). These applications demonstrate the increasing importance of attention-based models in pushing beyond the limits of traditional deep learning for automated breast cancer diagnosis.

Recent advancements in artificial intelligence (AI) have increasingly impacted applications within the Internet of Medical Things (IoMT), especially for automated breast cancer diagnosis. Early research centered on hybrid CNN–CNN-Transformer architectures to combine local feature extraction with global contextual understanding. For instance, a framework based on CrossViT combined with a Modified Growth Optimizer (MGO) for feature selection was tested on INbreast, MIAS, and MiniDDSM datasets, resulting in modest accuracy improvements (1.59%, 5%, and 0.79%, respectively) over existing benchmarks (43). This study underscored the potential of optimized feature selection to boost diagnostic accuracy in IoT-enabled clinical systems.

In histopathology, deep learning–based computer-aided diagnosis (CAD) systems have been developed to address challenges in manual examination. Standard CNNs often struggle with capturing global dependencies and lack interpretability, limiting their clinical usefulness. Vision Transformer (ViT) models were introduced for the benign–malignant classification task on the BreakHis dataset, leveraging self-attention mechanisms to model long-range interactions. The integration of explainable AI tools, including Grad-CAM, Integrated Gradients, Saliency Maps, LIME, and Attention Maps, allowed transparent prediction interpretation, outperforming CNNs and improving interpretability (44).

Building on these results, a multi-stage ViT framework was proposed to first distinguish benign from malignant histopathology images, then classify subtypes (45). Using 9, 109 BreakHis images, the method employed ViT/P16/224 and ViT/P32/384 models, reaching 98.99% accuracy with high F1 scores across various magnifications. This demonstrates the potential of hierarchical transformer models to enhance diagnostic precision in histopathology.

Transformer-based techniques have also been extended to subtype detection and patient-level predictions. A deep learning framework evaluated on a total of 111 whole-slide images with over 28, 000 annotated patches could detect cancer regions, classify subtypes (e.g., ductal vs. lobular carcinoma), and aggregate patch predictions to WSI-level outcomes (46). Comparing models like DenseNet-201, MobileNetV2, an ensemble, and ViT, the ViT-based system achieved 96.74% accuracy for cancer detection and 89.78% for subtyping. Majority voting further improved WSI-level performance, indicating transformer models’ feasibility for practical pathology workflows.

In mammography, transformer-based detection systems have been created to address dense breast tissue and subtle lesion features. The TransYOLO-GJO model integrated transformer attention into YOLOv9 and used the Golden Jackal Optimization algorithm for hyperparameter tuning. Tested on the CBIS-DDSM dataset, it achieved 95.1% mean Average Precision (mAP) and a 93.2% F1-score, with interpretability supported by Grad-CAM and SHAP visualizations (47).

Hybrid CNN–Transformer models like CTNet have addressed multimodal classification by combining channel attention, cross-attention, and dynamic attention mechanisms to enhance features from diverse imaging sources. On histology, mammography, and multimodal datasets, CTNet achieved 99%, 93%, and 86% accuracy, respectively, outperforming EfficientNet and DenseNet using simple feature fusion (48). Similarly, the InceptionNext-Transformer, with a four-stage design combining InceptionNext blocks and self-attention, recorded high accuracy up to 100% on the BreakHis binary classification dataset across seven different datasets (14).

Efforts to improve efficiency in whole-slide image analysis include lightweight transformer models such as B-EANet. It combines Vision Transformers with external attention to capture cross-sample dependencies while maintaining linear computational complexity. On datasets like BreakHis, CAMELYON 2017, and BACH 2018, B-EANet achieved outstanding results, including a 96.2% F1-score on BreakHis and low memory usage (4.35 MB), making it suitable for resource-limited clinical settings (49).

Self-supervised learning with transformer architectures is also being explored to reduce reliance on large, annotated datasets. Vision Transformers effectively model long-range dependencies, supporting tasks like segmentation, detection, and classification. However, issues of generalizability, external validation, and interpretability remain, requiring multi-institutional datasets and explainable frameworks (50).

In mammography, refined Vision Transformers (ViT-B32) have been used to classify X-ray images as malignant, benign, or normal. Tested on around 7, 000 images, these models reached accuracy and F1-scores between 91% and 94%, surpassing traditional CNNs like VGG16, MobileNet, Xception, and ResNet50 (51). Other models, such as MAX-ViT with gated attention and Harris Hawks optimization for feature selection, achieved 98.2% accuracy and 98.9% AUC on the King Abdulaziz University Mammogram Dataset (52). MammoViT, combining ResNet50-based hierarchical feature extraction with Vision Transformers capturing long-range dependencies, reached 97.4% accuracy across four BIRADS categories and addressed class imbalance with SMOTE (12).

In lesion segmentation, transformer models like TEBLS use hierarchical Vision Transformers with multi-scale fusion to capture local and global features, scoring an average DSC of 81.86% and AUC of 97.72% on CBIS-DDSM (53). Other hybrid methods embed a pretrained ViT within a UNet framework, enhanced with depth-wise separable convolutions to lessen computational load and overfitting, effectively improving segmentation performance (54).

Overall, these studies highlight the expanding role of transformer-based and hybrid deep learning models in breast cancer detection, classification, and segmentation. Although they improve accuracy, interpretability, and multi-modal integration, challenges remain regarding generalizability, computational efficiency, and dataset variability, pointing towards future research and clinical adaptation. **Table 8** shows a comparison between state-of-the-art techniques for Transformer-based and Hybrid Deep Learning Models for Breast Cancer detection.

**Table 8:** Comparison between state-of-the-art techniques for transformer-based and hybrid deep learning models for breast cancer diagnosis.

Ref	Technique/model	Dataset	Key metrics	Pros	Cons
(43)	CrossViT + Modified Growth Optimizer (MGO)	INbreast, MIAS, MiniDDSM	Accuracy gains: 1.59% (INbreast), 5% (MIAS), 0.79% (MiniDDSM)	Optimized feature selection in a hybrid CNN-Transformer model that is appropriate for IoMT	Some datasets are slightly improved; interpretability is unclear
(12)	Vision Transformer (ViT)	BreakHis	Accuracy: outperformed CNN baselines	Captures long-range dependencies; interpretable; explicable using LIME, Grad-CAM, etc.	Need a lot of data and are more expensive to compute than CNNs
(45)	Multi-stage ViT (ViT/P16/224 & ViT/P32/384)	BreakHis (9, 109 images)	Accuracy: 98.99%; high F1 scores	Excellent results at all magnifications; hierarchical categorization	Multistage design that is complex and computationally demanding
(46)	ViT-based framework for WSI cancer detection & subtype classification	111 WSIs; 28, 428 annotated patches	Patch-level: 96.74% (detection), 89.78% (subtype); WSI-level via majority voting	Enhances WSI-level accuracy by managing subtype classification	Annotation of large patches is necessary; patch aggregation increases complexity.
(51)	TransYOLO-GJO (YOLOv9 + Transformer + GJO)	CBIS-DDSM	mAP: 95.1%, F1-score: 93.2%	Optimized hyperparameters; interpretable with Grad-CAM & SHAP; capable of handling dense mammograms	Mammography was the focus, with little attention paid to other modalities.
(48)	CTNet (CNN–Transformer with Channel/Cross/Dynamic Attention)	Histology, Mammography, Multimodal	Accuracy: 99% (histology), 93% (mammography), 86% (multimodal)	Attention-driven feature refinement and efficient multimodal integration	Simple fusion limit complex modality interactions
(14)	InceptionNext-Transformer	7 datasets (histopathology, mammography, ultrasound)	Accuracy: 100% (BreakHis binary), ~99.97% (mammography)	Effective multi-scale feature integration and strong generalization	High model complexity; requires a lot of training
(50)	B-EANet (ViT + external attention)	BreakHis, CAMELYON 2017, BACH 2018	F1-score: 96.2% (BreakHis), high AUC across datasets	Lightweight; low memory footprint (4.35 MB); efficient WSI processing	restricted to patch-based WSI; inadequate patch aggregation may result in the loss of global context
(51)	SSL + Vision Transformers	Multiple breast imaging tasks	Varies by task	Captures global dependencies and lessens reliance on annotated data	Challenges in generalization and external validation
(52)	ViT-B32 (Refined ViT for X-ray)	~7, 000 mammography images	Accuracy & F1-score: 91–94%	Outperforms CNNs; applicable for mammographic analysis	Single modality (X-ray) focus; need larger datasets
(48)	MAX-ViT + GAFM + Harris Hawks Optimization + XGBoost	King Abdulaziz University Mammogram Dataset (BI-RADS)	Accuracy: 98.2%, AUC: 98.9%	Optimized feature fusion; strong discriminative learning	Computational overhead from MAX-ViT + HHO
(12)	MammoViT (ResNet50 + ViT)	Mammography (4 BIRADS categories)	Accuracy: 97.4%	Addresses class imbalance (SMOTE); captures local & global features	Restricted to mammography; intricate hybrid architecture
(53)	TEBLS (Transformer-based lesion segmentation)	CBIS-DDSM	Mean DSC: 81.86%, AUC: 97.72%	Captures local and global features; explainable	Segmentation-focused; may need a large amount of GPU RAM
(54)	ViT + UNet (Depthwise Separable Convolutions)	Breast tumor images	High segmentation accuracy (DSC not specified)	Effective training, global context through ViT, and decreased overfitting	Dependent on pretrained ViT; can be complex to implement

The comparative analysis presented in **Table 8** highlights the strong potential and adaptability of transformer-based and hybrid Transformer models across a variety of breast cancer diagnostic tasks, including lesion segmentation, classification, subtype detection, and whole-slide image analysis. Across multiple imaging modalities, including histopathology, mammography, and multimodal datasets, Vision Transformer (ViT) architectures, either as standalone models or integrated with convolutional backbones, have frequently demonstrated promising performance, often outperforming conventional CNN-based methods on the evaluated benchmark datasets in terms of accuracy, F1-score, AUC, and Dice similarity coefficient. Furthermore, ViT-based models reported high diagnostic performance on both large, complex datasets (e.g., CBIS-DDSM and CAMELYON 2017) and smaller benchmark datasets (e.g., BreakHis and MIAS), indicating their broad applicability across diverse breast cancer imaging tasks.

Although many transformer-based and hybrid deep learning models report impressive performance, these results should be viewed with caution. As shown in **Table 8**, some studies reach diagnostic accuracies of 98–99%, and nearly 100% on datasets like BreakHis and CBIS-DDSM. Yet, these outcomes mainly stem from retrospective datasets in controlled conditions and may be affected by issues like overfitting, class imbalance, limited dataset diversity, internal validation bias, and data leakage. Additionally, many studies depend on single-center or publicly available datasets without independent external validation, which can inflate perceived real-world performance. Therefore, high accuracy on benchmark datasets does not automatically mean the model is ready for clinical use. Future research must include external validation with independent multicenter cohorts, prospective clinical trials, calibration checks, robustness testing across different patient groups and imaging devices, and compliance with regulatory and reporting standards before deploying transformer-based AI in breast cancer diagnosis.

Transformers have demonstrated exceptional efficacy in modelling long-range spatial dependencies and global contextual relationships across a variety of use cases. These relationships are essential for identifying subtle tumor boundaries in dense breast tissue, differentiating histological subtypes, and combining patch-level predictions into trustworthy whole-slide image decisions. By fusing the global attention processes of transformers with the localized feature extraction of CNNs, hybrid frameworks further enhanced these benefits, resulting in increased computing efficiency, robustness, and interpretability. By facilitating transparent interpretation of model predictions, a crucial prerequisite for practical implementation in IoMT-enabled healthcare systems, the integration of explainable AI approaches, such as attention mapping, Grad-CAM, and SHAP, has further improved clinical credibility.

**Figure 12** illustrates the temporal evolution of major artificial intelligence methodologies in breast cancer imaging research between 2020 and 2026. The results demonstrate that convolutional neural networks (CNNs) dominated earlier studies, particularly between 2020 and 2022, reflecting their foundational role in AI-based medical image analysis. However, transformer-based architectures, including Vision Transformers (ViT) and hybrid CNN–Transformer frameworks, have shown substantial and continuous growth since 2022, indicating a clear methodological transition toward attention-based deep learning systems. Additionally, explainable artificial intelligence (XAI) techniques exhibited progressive adoption over time, especially during 2024–2025, highlighting the increasing research emphasis on model interpretability, transparency, and clinical trustworthiness. Overall, the findings quantitatively confirm the growing adoption of transformer-driven and explainable AI methodologies within breast cancer diagnostic research.

**Figure 12:**
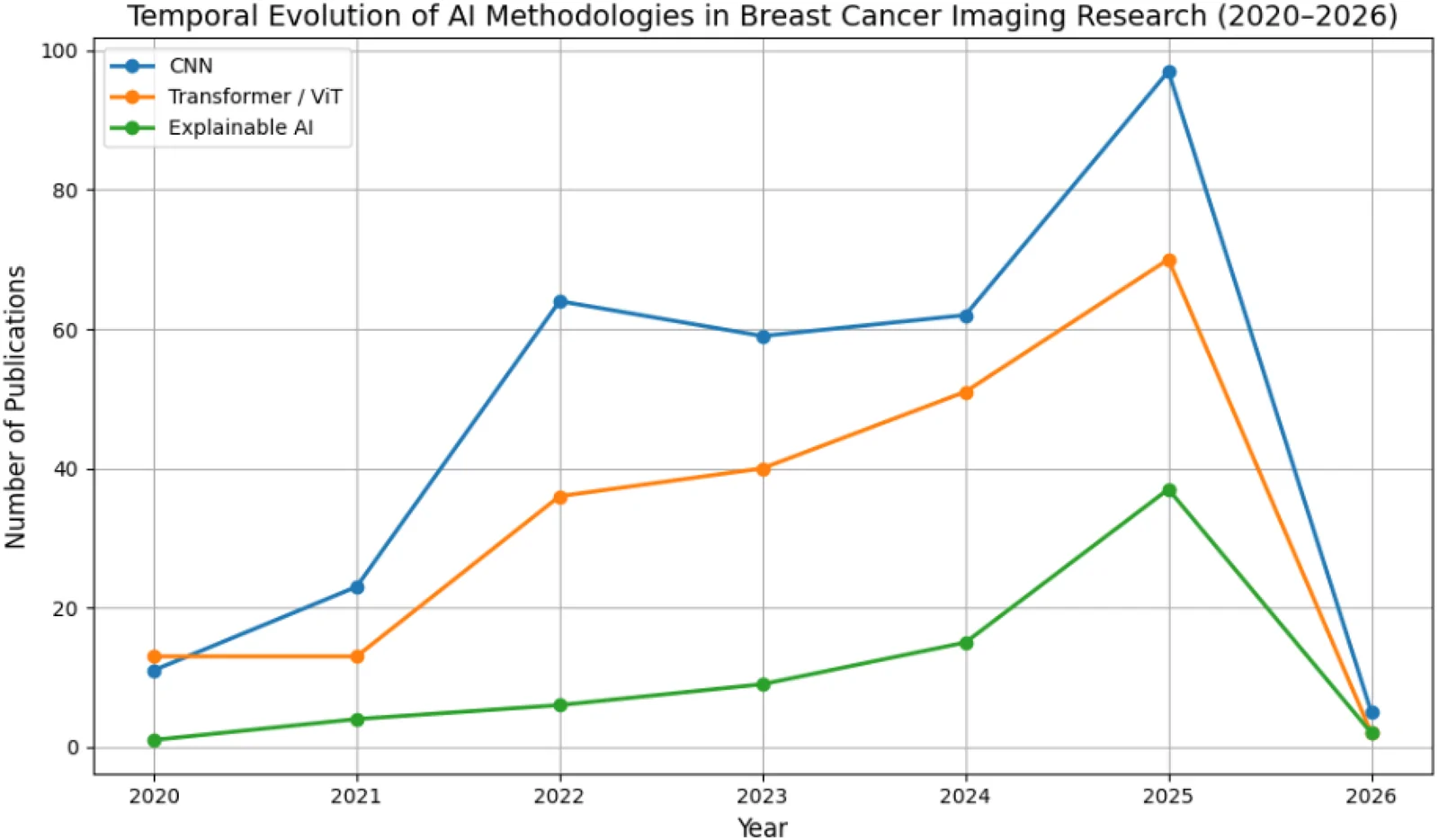
Temporal evolution of CNN-, Transformer-, and explainable AI-related methodologies in breast cancer imaging research between 2020 and 2026.

From a dataset perspective, transformer-based models showed resilience to class imbalance and limited data availability when paired with methods like self-supervised learning, data augmentation, and feature optimization algorithms. They also showed adaptability to different annotation granularities, ranging from image-level labels to pixel-level segmentation masks. These models might be used in clinical settings with limited resources without compromising diagnostic performance, thanks to lightweight transformer variations that further addressed computational limitations.

## 6 Explainable AI, or XAI

The bibliometric study demonstrated a distinct shift in research priorities toward AI systems that are clinically reliable and explainable. Explainable Artificial Intelligence (XAI) is one of the fastest-growing research hotspots, according to keyword co-occurrence, temporal evolution, and burst analyzes. This finding reflects the increased emphasis on model transparency, interpretability, and clinical adoption. To close the gap between strong predictive performance and clinical trust, this section examines the most popular XAI algorithms, their underlying theories, and their applications in breast cancer detection.

Machine learning (ML) and deep learning (DL) are two types of AI technology used in healthcare applications. Numerous internal hidden layers account for the term “deep. “Large data sets and powerful workstations are necessary for these models. Nevertheless, both methods are perceived as “black boxes” that fail to provide an explanation for the predictions (55, 56).

Despite that AI-based systems have demonstrated promise in helping with breast cancer diagnosis and screening, one of the main causes of the low number of AI tool implementations and integrations in clinical practice can be attributed to the lack of understanding and transparency in predictions, even though these systems typically exhibit good accuracy. Explainable AI, or XAI as it is more widely known, has been proposed as a solution to improve the performance and transparency of deep learning models in the medical field. To effectively implement XAI in medical contexts, numerous frameworks and techniques have been developed (55, 56).Explainability is the ability to provide an understandable explanation for a machine learning model and its outcomes. Explainability, according to some literature, is the idea that a machine learning model and its conclusions can be explained to a person in terms that make sense (57).

The medical team can better comprehend the AI model’s choice and visual explanation thanks to XAI’s ability to identify significant areas in medical imaging (58). Some of the most well-known XAI techniques include Local Interpretable Model-agnostic Explanations (LIME), SHapley Additive exPlanations (SHAP), Gradient-weighted Class Mapping (GradCAM), Grad-CAM++, Integrated Gradients, and Attention-based methods, which are frequently employed in a variety of application areas to decipher and comprehend the predictions made by deep learning and machine learning models. Here, we contribute to expanding the present understanding of XAI and its use in healthcare, particularly regarding breast cancer.

Depending on the type of explanation and the part of the model they are intended to interpret, XAI techniques can be broadly classified. **Table 9** summarizes the differences between these groups in terms of their focus, supporting tools, visualization methods, and suitable model families. Additionally, a lot of XAI tools are made especially for feature attribution, which allows for a thorough comprehension of model behavior at the input level (59). **Table 10** provides a thorough categorization of popular XAI tools based on these dimensions, making it easier to compare and choose suitable explainability strategies for various models and application scenarios.

**Table 9:** XAI category.

XAI category	Definition	Focus	Tools	Figures	Models	Using
1)Feature Attribution	measure each input feature’s contribution to a model’s prediction, allowing for both local and global interpretability.	Feature Contribution	SHAP, LIME, DeepLIFT, ELI5, Integrated Gradients	Summary plots, Bar plots, Force plots, Waterfall plots, Attribution Heatmaps	ML&DL	Medical decision support, Risk prediction, Feature validation
2)Visual XAI	visual explanation techniques provide spatial interpretability.	Spatial Regions	Grad-CAM, Grad-CAM++, EigenCAM, Attention heatmaps, SmoothGrad, Guided Backpropagation	Heatmaps, Saliency maps, Activation maps, Overlay maps	CNN, Vision Transformers	Histopathology, Mammography, Ultrasound, MRI/CT
3)Framework Libraries	provide unified toolkits that combine several explainability methodologies.	Tool Integration	Captum(PyTorch), TensorFlow Explain, InterpretML, OpenXAI	Heatmaps, Bar charts, Attribution plots, Shape functions	ML&DL PyTorch/TensorFlow/Scikit-learn	Feature attribution, Gradient-based explanations, Global&Local explanations, Unified APIs
4)Production XAI	Production-level Explainability is expanded by XAI systems to encompass governance, fairness, and lifecycle monitoring in practical applications.	Deployment&Governance	Google XAI, IBM OpenScale	Feature attribution, Bias detection, Model monitoring, Explainability dashboards	____	Bibliometric studies, Evolution of XAI, AI governance

**Table 10:** XAI tool categorizes.

XAI tools	Global explainability	Local explainability	Model-agnostic XAI	Model-specific XAI	*Post-hoc* explainability	Feature attribution	Visual explanation	Interpretable
Definition	how an AI model generally makes decisions over the whole dataset	describe why a model chose a particular course of action for a given instance	applicable to various AI architectures.	produce explanations by utilizing the inherent mechanisms of unique AI models.	explain the model predictions after the training and inference processes	XAI techniques generate numbers or values for them.	A form of explainability based solely on visual explanations.	describes the use of white box models, which are inherently interpretable.
SHAP(Global)	✔		✔		✔	✔		
SHAP(Local)		✔	✔		✔	✔		
LIME		✔	✔	✔	✔	✔		
Grad-CAM		✔		✔	✔		✔	✔
Partial Dependence plot (PDP)	✔		✔				✔	
Permutation Importance	✔		✔					
Explainable Boosting (EBM)	✔							✔
Anchors		✔						
Counterfactual Explanations		✔	✔					
Integrated Gradients				✔		✔		
Attention		✔		✔			✔	✔
LRP				✔				
Decision Trees	✔							✔
Linear/Logistic Regression								✔
Rule-based models	✔							✔
DeepLIFT	✔	✔			✔			

Model-agnostic XAI tools are suitable for a variety of machine learning and deep learning algorithms since they are made to offer explanations without depending on the internal structure of the underlying model (57, 60–62). These tools are compared according to important factors such as supported model families, explanation outputs, visual explanation forms, and input data kinds, as shown in **Table 11**.

**Table 11:** XAI model-agnostic.

XAI tools	Input type	Figures	Models	Output
LIME	Tabular/Text/Image	Bar plots-Superpixels	ML&DL	Feature importance (Local)
SHAP	Tabular/Text/Image	Summary, Dependence, Force, Waterfall	ML&DL	SHAP values
Permutation Importance	Tabular	Bar plot	ML&DL	Feature importance (Global)
Partial Dependence plot (PDP)	Tabular	Line/2D plot	ML&DL	Feature-Prediction relation
Individual Conditional Expectation (ICE)	Tabular	Line plots	ML&DL	Individual feature effect
Anchors	Tabular/Text/Image	Rule-based figures	ML&DL	IF-THEN rules
Counterfactual Explanations	Tabular	Tables/Plots	ML&DL	Minimal feature changes

After classifying XAI tools based on the standards shown in the preceding **Table 11**, we move on to provide a detailed explanation of the most significant and popular methods. The most well-known and widely used XAI tools are covered in detail in the following section (62).

### 6.1 SHapley additive explanations

An open-source Python package called SHAP is used to decipher and interpret machine learning and deep learning model predictions. SHAP is an explainable machine learning model that comes from cooperative game theory’s Shapley values. SHAP values give both a global and local understanding of the model by measuring the influence of each feature in a particular data instance on the model’s output. SHAP explanations are dependable and mathematically solid since this method ensures crucial theoretical characteristics like local precision, consistency, and missingness (61–65).

### 6.2 Gradient-weighted class mapping

A popular explainability method for interpreting the choices made by deep learning models in computer vision, especially convolutional neural networks (CNNs), is Grad-CAM. Grad-CAM is a local backpropagation-based technique that generates a coarse localization class-specific heatmap with the hot portion corresponding to a certain class using the feature maps generated by a CNN’s final layer (55, 64).

The library supports several sophisticated variations in addition to the conventional Grad-CAM, including Grad-CAM++, Score-CAM, XGrad-CAM, Ablation-CAM, Layer-CAM, HiRes-CAM, FullGrad, and Eigen-CAM (65, 66).

### 6.3 Local interpretable model-agnostic explanations

The fundamental idea of LIME is to fit a simpler, more comprehensible surrogate model, such as a weighted linear regression, decision tree, or sparse linear model, to perturb samples to approximate the complex model locally around a particular prediction. LIME generates explanations by repeatedly calling the model with perturbed inputs and learning a local surrogate model around the instance of interest. This protocol is implemented by the standard Python module lime, which is accessible through PyPI. It supports many explainers, including LimeTabularExplainer for tabular datasets, LimeTextExplainer for text classifiers, and LimeImageExplainer for image classification jobs. The trained model to explain, a test instance (such as a feature vector for tabular data, a text document, or an image), and the training data used to determine the perturbation distribution are the usual inputs to LIME. LIME perturbs the data surrounding the input and trains the local surrogate model using the model’s predicted outputs on those perturbed samples (55, 59, 61, 62, 64, 67).

### 6.4 Attention visualization

A XAI method called Attention Visualization is mostly utilized in Transformer-based models and deep learning to display the portions of the input data that are most important during prediction. In Natural Language Processing (NLP), it displays the words or tokens that have the most effect on the model’s decision, whereas in image processing, it emphasizes significant picture areas. By displaying attention weights as heatmaps or graphical attention maps, attention visualization aids users and researchers in comprehending model behavior, enhancing transparency, identifying biases, and boosting confidence in AI systems (68).

### 6.5 Saliency map

Saliency Map (69), is used to determine which aspects of an input are most crucial for influencing a deep learning model’s prediction. By calculating the gradient of the output with respect to the input picture, saliency maps in image classification tasks emphasize the pixels or areas that have the most influence on the final choice. Researchers and users can more easily comprehend how the model concentrates on particular traits when the highlighted areas are shown as heatmaps. In computer vision applications like medical imaging, object identification, and image recognition, saliency maps enhance model transparency and aid in identifying improper model behavior.

After The study of 608 references can yield obvious insights when the most significant Explainable Artificial Intelligence (XAI) methodologies have been reviewed and explained in depth. The findings indicate that the most popular XAI method was general Explainable AI and interpretability methods (31.91%), followed by Grad-CAM (14.64%), which was extensively used in medical imaging and deep learning research because of its capacity to identify significant picture areas that affect model predictions. Of the examined studies, Saliency Maps accounted for 5.92% and CAM-based techniques for 11.84%. Furthermore, 3.95% of the references mentioned SHAP, 2.96% mentioned LIME, and 1.97% mentioned Attention Visualization approaches. However, with just 0.99% of the examined references, Integrated Gradients was shown to be the least popular XAI approach are categorized in **Table 12**. These results show that XAI techniques focused on visualization continue to be the most popular option in current AI research, particularly in computer vision applicati.

**Table 12:** Statistical analysis of XAI techniques used in 608 references.

XAI method	Percentage%	References count
general Explainable AI	31.91%	33
Grad-CAM	14.64%	19
CAM	11.84%	12
Saliency Maps	5.92%	8
SHAP	3.95%	7
LIME	2.96%	3
Attention Visualization	1.97%	3
Integrated Gradients	0.99%	1

XAI is one of the fastest-growing research areas, according to the bibliometric study, which reflects the rising need for transparent, understandable, and reliable AI systems in healthcare. In line with this pattern, the systematic review shows a significant rise in the use of XAI techniques, with more recent research utilizing techniques like Grad-CAM, SHAP, LIME, Integrated Gradients, and attention-based approaches to enhance the interpretability of deep learning models without sacrificing diagnostic performance. These methods have grown increasingly common, as **Table 12** illustrates, underscoring the rising understanding that explainability is a requirement for therapeutic acceptability. However, rather than fully integrated decision-support frameworks, their utilization is still mostly concentrated on *post hoc* visualization and feature attribution. Additionally, the reviewed research shows a deficiency of clinically approved explainability criteria and structured evaluation methodologies, which restricts the objective evaluation of clinical value and explanation quality. As a result, even though XAI has become a major area of research, it should be seen as a necessary prerequisite for establishing clinician confidence, facilitating regulatory approval, and enabling the successful clinical translation of AI-based breast cancer diagnosis rather than just an optional improvement.

## 7 Hyperparameter optimization

The bibliometric findings also demonstrated an increasing shift away from traditional deep learning methods and toward more advanced transformer-based and hybrid models. The importance of hyperparameter optimization (HPO) in enhancing model performance, resilience, computing economy, and generalizability is highlighted by this growing methodological complexity. Inspired by these findings, this section examines the most popular HPO techniques and talks about how they improve AI models for diagnosing breast cancer.

AI is pervasive in daily life these days. To maximize the data, ML algorithms and their parameters need to be cleverly set up. Hyperparameters are those parameters that must be set before the algorithm is trained. Hyperparameters are predetermined and govern the learning process itself, in contrast to model parameters (such as weights and biases), which are learnt during training. To guarantee the optimal performance of machine learning (ML) algorithms, HPO is an essential step. The popularity of deep learning methods, which have demanding features (e.g., the requirement for enormous amounts of data and time to train the models, high model complexity, and a broad mix of hyperparameter types), has likely led to an increase in interest in HPO in recent years. The primary hyperparameter optimization techniques are categorized in **Table 13** based on their search behavior, optimization strategy, computational cost, and balance between exploration and exploitation (70–73).

**Table 13:** The hyperparameter optimization techniques are categorized as.

No.	HPO Category	HPO Method	Description	
1	Exhaustive and Deterministic Methods	Grid Search	• Evaluates every feasible set of values for the predetermined hyperparameters. • Ensures that the optimal solution can be found inside the grid. • costly to compute and unfeasible for high-dimensional spaces.	
		Manual Search	• Expert knowledge is used to adjust hyperparameters. • Simple, but not scalable and subjective.	
2	Randomized Methods	Random Search	• Hyperparameter values are randomly selected from predetermined distributions. • more effective in high-dimensional spaces than grid search. • widely employed as a standard HPO technique.	
3	Bayesian Optimization Methods	Bayesian Optimization	• creates a surrogate model based on probability. • balances exploration and exploitation by using an acquisition function. • Effective for costly black box bjective functions. • Common Techniques • Tree-structured Parzen Estimator (TPE) (used in Hyperopt, Optuna) • SMAC (Sequential Model-based Algorithm Configuration) • Gaussian Process Optimization	
4	Gradient-Based Optimization	Gradient-Based HPO	• calculates hyperparameter gradients in relation to validation loss. • Effective for hyperparameters that are continuous. • depends on the model and needs to be differentiable.	
5	Evolutionary and Metaheuristic Methods	Genetic Algorithms (GA)	• Natural evolution-inspired population-based search. • Uses crossover, mutation, and selection.	• These techniques, which naturally strike a balance between exploration and exploitation, are categorized as metaheuristics.
		Particle Swarm Optimization (PSO)	• simulates how particles behave collectively. • Quick convergence but could be affected by early	
		Ant Colony Optimization (ACO)	• Pheromone trails are used to generate probabilistic paths.	
		Differential Evolution (DE)	• Effective in spaces with continuous parameters.	
6	Multi-Fidelity Optimization	Successive Halving	• gradually concentrates on configurations that show promise after allocating scarce resources to numerous configurations.	
		Hyperband	• integrates early halting with random search. • greatly lowers the cost of computing.	
7	Reinforcement Learning–Based Methods	RL-based HPO	• considers hyperparameter tuning as a task involving sequential decision-making. • uses agents to select hyperparameters in an adaptive manner.	
8	Multi-Objective Hyperparameter Optimization		• maximizes several goals at once (e.g., accuracy vs. training time). • Solved using: • Multi-objective EA based on decomposition (MOEA/D) • Pareto-based Bayesian Optimization • (Non-dominated sorting GA II (NSGA-II)	

### 7.1 Metaheuristic optimization algorithms

Metaheuristic algorithms are essential to hyperparameter optimization because they offer effective search strategies for intricate and high-dimensional hyperparameter spaces. Usually, hyperparameter optimization is presented as a black-box problem with an expensive objective function that lacks gradient information. Additionally, metaheuristic algorithms may balance exploration with exploitation as summarized in **Table 14**. thanks to mechanisms like selection, crossover, mutation, and adaptive update rules. This is crucial for finding reliable and almost ideal hyperparameter values. As a result, metaheuristic optimization has emerged as a popular and potent strategy for fine-tuning hyperparameters in deep learning and machine learning models, especially when conventional grid or random search techniques are unproductive or computationally inefficient (71).

**Table 14:** Exploration vs. exploitation.

Aspect	Exploration	Exploitation
Definition	• The process of looking for uncharted territory in the hyperparameter space is referred to as exploration. Increasing diversity and preventing premature convergence to less-than-ideal solutions are its primary objectives.	• The goal of exploitation is to improve and refine ideas that are already promising by conducting local searches in high-performance areas.
Characteristics	• encourages a variety of potential solutions • lowers the possibility of local optima • promotes global search	• Focus on local optimization • speeds up convergence • enhances fine-grained performance
Examples of processes for	• Random sampling • High-variance Bayesian sampling • Diversification of populations in metaheuristics • Genetic algorithms with mutation operators	• Local search • Crossover operators • Greedy selection • Bayesian posterior updates
• Exploration and exploitation must be balanced in a well-crafted optimization system. While excessive exploitation increases the chance of becoming stuck in local optima, excessive exploration slows convergence. This harmony is essential to • Metaheuristic algorithms • Bayesian Optimization • Reinforcement learning-based HPO		

Metaheuristic algorithms are a collection of methods for resolving complicated, vast search space, and non-convex optimization problems to which HPO problems belong. The essential foundation of metaheuristic optimization algorithms is the harmony between exploration and exploitation. Exploitation concentrates on stepping up the search around viable solutions that have already been found, allowing for incremental gain in performance. For example, selection, crossover, and local search strategies improve exploitation by maintaining and integrating high-quality solutions, while mutation and random perturbations mainly help exploration by providing diversity. The capacity of a metaheuristic algorithm to dynamically balance these two complementing processes is crucial to its efficacy, especially in high-dimensional, non-convex optimization issues like hyperparameter optimization unbalanced approach might result in either excessive exploitation, which causes premature convergence, or excessive exploration, which causes sluggish convergence. As a result, it is commonly believed that the exploration-exploitation trade-off is the fundamental idea guiding the effectiveness of metaheuristic optimization methods (72).

**Table 14** provides a clear and structured comparison between exploration and exploitation in the context of hyperparameter optimization (HPO) and metaheuristic algorithms. To effectively explore and exploit the hyperparameter search space, metaheuristic algorithms especially evolutionary algorithms use genetic operators like crossover and mutation as summarized in **Table 15** (74).

**Table 15:** Role of crossover & mutation in HPO.

Aspect	Crossover (Exploitation)	Mutation (Exploration)
Technique	Hyperparameters from two or more high-performing configurations (parents) are combined by the crossover operator to produce new candidate solutions (offspring). Crossover encourages exploitation in hyperparameter optimization by mixing optimal hyperparameter values from several models.	Mutation introduces low-probability random perturbations to particular hyperparameters. By preserving diversity and introducing randomness into the population, mutation improves exploration.
Function	• Uses the search space’s promising regions. • Transfers helpful patterns of hyperparameters. • Speeds up the convergence of ideal setups.	• Extends the search to previously untapped areas. • keeps local optima from becoming stagnant. • presents new values for the hyperparameter.
Example	Combining the best batch size from one model with a high learning rate from another.	varying the number of neurons or dropout rate at random.

### 7.2 Hyperparameter optimization tools

In contemporary machine learning, hyperparameter optimization has emerged as a key component that bridges the gap between fully automated model improvement and manual tuning as summarized in **Table 16**. Optuna is one of the most well-liked HPO tools because of its straightforward Python interface, early stopping pruning, TPE optimization, and easy connection with frameworks like PyTorch and TensorFlow. Ray Tune is perfect for GPU clusters because it is designed for large-scale distributed experiments and supports population-based training, HyperBand, ASHA, and Bayesian optimization.The amount of control needed, framework compatibility, and computing scale all play a role in selecting the best HPO tool (72, 74–77).

**Table 16:** Overview of tools for hyperparameter optimization (HPO).

No.	Tools		Features	Use case	Optimization	Language	URL of tools
1	HPO	Optuna	Uses TPE, visual dashboards, PyTorch/LightGBM/TensorFlow, pruning	General-purpose HPO, flexible experiments	random search (RS), TPE, NSGA-II, Covariance matrix adaptation evolution strategy (CMA-ES), grid search (GS).	Python	GitHub - optuna/optuna: A hyperparameter optimization framework
2		Ray Tune	Bayesian optimization, Population-based training (PBT), Asynchronous Successive Halving Algorithm (ASHA) HyperBand	Large-scale deep learning experiments	GS, Optuna, Evolutionary algorithms (EA), Nevergrad, RS, Bayesian optimization (BO)	Python	https://docs.ray.io/en/latest/tune/index.html
3		Hyperopt	TPE-based, lightweight	Tabular ML or classic pipelines	AdaptiveTPE, TPE, RS	Python	https://hyperopt.github.io/hyperopt/
4		Scikit-Optimize (skopt)	Bayesian search, scikit-learn style	Academic experiments, interpretability	GS, RS, Gaussian process (GP)	Python	https://github.com/scikit-optimize/scikit-optimize
5		KerasTuner	HyperBand, Random search, Bayesian optimization, TensorFlow/Keras	Keras/TensorFlow deep learning	RS, BO, Hyperband	Python	https://github.com/keras-team/keras-tuner
6		Google Vizier	Multi-metric, black-box optimization, early stopping	Industrial, high-end tuning	RS, GS, BO	Python, C++, Golang	https://cloud.google.com/vertex-ai
7		BoTorch	Custom Bayesian optimization, constraints, multi-objective	Research and custom objectives	GP	Python	GitHub - meta-pytorch/botorch: Bayesian optimization in PyTorch
8		FacebookAx	Custom Bayesian optimization, constraints, multi-objective	Research and custom objectives	GP, BoTorch	Python	https://github.com/facebook/Ax
9		BayesianOptimization	Sample-efficient,	Deep Learning, Hyperparameter tuning, black box	GP, TPE	Python	https://github.com/bayesian-optimization/BayesianOptimization
10		OpenBox	Bayesian Optimization, Multi-fidelity, multi-objective, constraints	AutoML pipelines, Industrial ML systems	BO, multi-objective maximum entropy search (MESMO), Hyperband	Python	https://github.com/PKU-DAIR/open-box
11		DEAP	Highly flexible, customizable	Feature selection, Neural architecture evolution	PSO, GA, CMA-ES, NSGA-II, NSGA-III	Python	https://github.com/DEAP/deap
12		EvoTorch	Scalable with PyTorch, distributed optimization	Reinforcement Learning, Neuroevolution	evolutionary	Python	https://github.com/nnaisense/evotorch
13		BayesOpt	Bayesian Optimization, Gaussian Process, continuous & discrete variables, mathematical foundation	Scientific optimization, Expensive simulations Robotics and control systems	GP	Python	https://github.com/rmcantin/bayesopt
14		Nevergrad	Gradient-free optimization, stochastic functions	Black-box optimization, Reinforcement learning problems, Hyperparameter tuning	GA, RS, BO, PSO	Python	https://github.com/facebookresearch/nevergrad
15		jMetal(Py)	Multi-objective optimization, benchmarking support	Engineering optimization, Trade-off analysis	GA, ES, NSGA-II, MOEA/D	Python, Java	https://github.com/jMetal/jMetalPy
16		Goptuna	Bayesian optimization, pruning strategies	Neural network tuning, Research experiments, Deep learning HPO	TPE, RS, ASHA	Golang	https://github.com/c-bata/goptuna
17		Mango	Bayesian optimization, Lightweight, parallel execution	Academic experiments, Fast prototyping	BO	Python	https://github.com/ARM-software/mango
18		Gradient-Free Optimizers (Hyperactive)	No derivatives required, noisy objectives	Black-box ML models, Simulation-based optimization, Hyperparameter tuning	PSO, RS, GS, BO,	Python	https://github.com/SimonBlanke/Gradient-Free-Optimizers
19	AutoML	Microsoft NNI (Neural Network Intelligence)	Feature engineering, HPO, model compression, NAS, GUI	Teams needing automation and customization	EA, GS, PBT RS, Hyperband, BO	Python	https://github.com/microsoft/nni
20		MicrosoftFLAML	Lightweight AutoML, classical ML, fast optimization	Industrial AutoML, Tabular data problems	GS, RS, Optuna	Python,.NET	https://github.com/microsoft/FLAML
21		OracleAutoML	Cloud-based AutoML,	Business analytics, Cloud ML deployment, large enterprise datasets	gradient-based	Python	https://docs.oracle.com/en/database/oracle/machine-learning/
22		MicrosoftAzureML AutoML	Cloud scalability, Built-in model explainability	Time-series & tabular tasks, Production ML systems	RS, BO, GS	Python	https://azure.microsoft.com/en-us/solutions/automated-machine-learning/
23		AutoKeras	NAS, Keras/TensorFlow, Automated model design	Deep learning, Image & text classification, Rapid DL prototyping	GP	Python	https://github.com/keras-team/autokeras
24		Auto-PyTorch	AutoDL with PyTorch, Bayesian optimization, performance on tabular data	Academic research, Benchmark, High-performance DL tuning	SMAC, Hyperband	Python	https://github.com/automl/Auto-PyTorch
25		Amazon SegeMaker HPO	Bayesian optimization, distributed training	Production ML pipelines	GS, Hyperband, RS, BO	Python, R	https://aws.amazon.com/machine-learning
26	NAS	MicrosoftArchai	Advanced NAS, Efficient model scaling	Efficient deep models, Neural architecture research, Edge AI	RS, evolution	Python	https://github.com/microsoft/archai
27		Katib	Bayesian, Random, Evolutionary search, Kubernetes-native HPO	Distributed deep learning, MLOps pipelines, Cloud-native ML	BO, Optuna, PBT, Hyperband, Hyperopt	Python	https://github.com/kubeflow/katib
28		DeepHyper	Asynchronous Bayesian optimization, Scalable AutoML & HPO	Scientific ML, Large-scale DL optimization, Supercomputing environments	Scalarization, BO	Python	https://github.com/deephyper/deephyper
29		NePS	Neural architecture, Bayesian optimization, Hierarchical configuration	Neural architecture search, Complex search spaces, AutoDL research	Hyperband, BO, PriorBand	Python	https://github.com/automl/neps
30		Hypernets	Modular architecture, AutoML framework for tabular data, Feature engineering + HPO	Tabular datasets, AutoML pipelines, Business ML problems	EA, NSGA-II MCTS, NSGA-III	Python	https://github.com/DataCanvasIO/Hypernets

The research was assessed using both HPO techniques and HPO tools based on the examination of 608 examined references. According to the findings, the most popular HPO strategy was Genetic Algorithms (1.97%), followed by Particle Swarm Optimization (1.32%), Bayesian Optimization (0.33%), and Grid Search (0.16%). The most popular HPO framework and tool was Bayesian Optimization (0.33%), followed by KerasTuner (0.16%) as summarized in **Table 17**. These results show that, compared to contemporary dedicated HPO frameworks and automated optimization platforms, classical optimization procedures are still more often documented in the literature.

**Table 17:** Overview of tools for hyperparameter optimization (HPO).

Aspect	HPO	Percentage%	References count
HPO Strategies	Genetic Algorithms	1.97%	12
	Particle Swarm Optimization	1.32%	8
	Grid Search	0.16%	1
HPO Tools	Bayesian Optimization	0.33%	2
	KerasTuner	0.16%	1

The bibliometric study demonstrated a distinct methodological trend toward more complex AI architectures, highlighting the rising significance of HPO for optimizing model resilience and performance. In line with these conclusions, the systematic review shows that HPO methods, such as Grid Search, Random Search, Bayesian Optimization, Optuna, Genetic Algorithms, Particle Swarm Optimization, and other metaheuristic approaches, can significantly increase model robustness, computational efficiency, convergence speed, and diagnostic accuracy in a variety of breast cancer applications. However, as **Table 17** summarizes, only around 15–25% of the examined studies explicitly described their hyperparameter tuning methodologies, indicating that HPO use and reporting are still uneven. The majority of research limit reproducibility, transparency, and fair comparison among models by reporting only the final performance measurements while leaving out crucial information about the search strategy, optimization procedure, and investigated parameter space. As a result, HPO is still underreported and inadequately standardized despite its proven advantages. All things considered, the combined bibliometric and systematic evidence shows that rigorous and transparent HPO is not only a performance-enhancement strategy but a basic prerequisite for creating dependable, repeatable, and clinically deployable AI systems for breast cancer detection.

## 8 Discussion and future directions

To maintain alignment between the study objectives, analytical methods, and reported findings, this section organizes the results using a research question–driven structure based on RQ1–RQ7. Alongside presenting the findings, the section also addresses the limitations related to each research question. Moreover, it goes beyond descriptive reporting by providing an integrated discussion and future-oriented perspective that identifies emerging research directions and highlights practical implications for the development of clinically applicable AI systems.

### 8.1 Research question–based analysis

To strengthen the methodological–result alignment and address the hybrid nature of the study, the results are now systematically organized according to the predefined research questions (RQ1–RQ7). This structure ensures that the bibliometric analysis is explicitly linked to quantitative evidence while integrating thematic interpretations derived from the reviewed literature.

RQ1. Field Evolution: Clinical Tasks, Modalities, and Methods

The evolution of AI-based breast cancer research demonstrates a clear transition from traditional machine learning approaches to advanced deep learning paradigms. Quantitative analysis (**Table 1**) shows that diagnosis dominates the literature (~71%), followed by prognosis (~27%), while treatment-response prediction remains underexplored. Imaging-based modalities, particularly mammography, continue to lead; however, recent years show an increasing shift toward multimodal integration and hybrid architectures, reflecting a more comprehensive clinical perspective.

RQ2. Explainable AI and Trustworthiness

The adoption of explainable AI (XAI) techniques has increased significantly in recent studies, though it remains incomplete. Analysis (**Table 12**) indicates that a growing proportion of studies incorporate methods such as Grad-CAM, SHAP, and LIME, primarily to enhance interpretability in deep learning models. While these techniques improve transparency and support clinical validation, their integration is often limited to visualization rather than full decision-support frameworks, indicating a gap between technical implementation and real-world clinical trust.

RQ3. Hyperparameter Optimization Practices

Hyperparameter optimization (HPO) remains inconsistently reported across the literature. Only approximately 15–25% of studies explicitly describe tuning strategies (**Table 17**), with methods including grid search, Bayesian optimization, and metaheuristic algorithms such as genetic algorithms and particle swarm optimization. Despite its importance for model performance, most studies report only final evaluation metrics without detailing search strategies or parameter spaces. This highlights a significant gap in reproducibility and methodological rigor.

RQ4. Datasets and Software Ecosystems

The analysis of datasets and computational tools reveals a strong dependence on a limited set of benchmark datasets. Imaging datasets such as CBIS-DDSM, MIAS, and INbreast are most frequently used, while large-scale genomic and population datasets are underutilized. Similarly, software frameworks (e.g., TensorFlow and PyTorch) are often implicitly used but rarely reported explicitly, indicating a lack of transparency in implementation details. This trend affects both methodological choices and reproducibility across studies.

RQ5. Algorithmic Trends and Emerging Architectures

Deep learning models dominate the field, significantly surpassing traditional machine learning approaches (**Figure 12**). Within deep learning, a notable trend is the rapid emergence of transformer-based architectures and attention mechanisms, particularly after 2021. These models are increasingly integrated with convolutional networks, forming hybrid architectures that improve performance and enable more effective feature representation.

RQ6. Mammography as a Core Modality

Mammography remains the central modality in AI-based breast cancer detection, accounting for approximately 60.7% of dataset usage. Over time, methodologies based on mammographic imaging have evolved from simple CNN-based classifiers to advanced architectures incorporating attention mechanisms, explainability tools, and multimodal fusion. Despite this evolution, the dominance of mammography highlights both its clinical importance and the relative underrepresentation of complementary modalities.

RQ7. Emerging Hotspots and Translational Potential

Burst detection and keyword growth intensity analysis identify several rapidly emerging research hotspots. Key topics include Explainable AI (XAI), transformer-based models, attention mechanisms, and multimodal learning, all of which exhibit strong recent growth. These areas demonstrate high translational potential, as they address critical challenges such as model interpretability, data integration, and clinical applicability. The findings suggest a shift toward clinically integrated AI systems that combine performance, transparency, and robustness.

### 8.2 Clinical AI translation pipeline

**Figure 13** illustrates the clinical AI translation pipeline for breast cancer diagnosis and screening, highlighting the progression from research development to real-world healthcare impact. The process begins with the identification of a clinical need, followed by AI tool design, model development, and internal validation using curated datasets. At this stage, the primary objective is to establish proof-of-concept performance and demonstrate the feasibility of applying artificial intelligence to clinically relevant diagnostic tasks.

**Figure 13:**
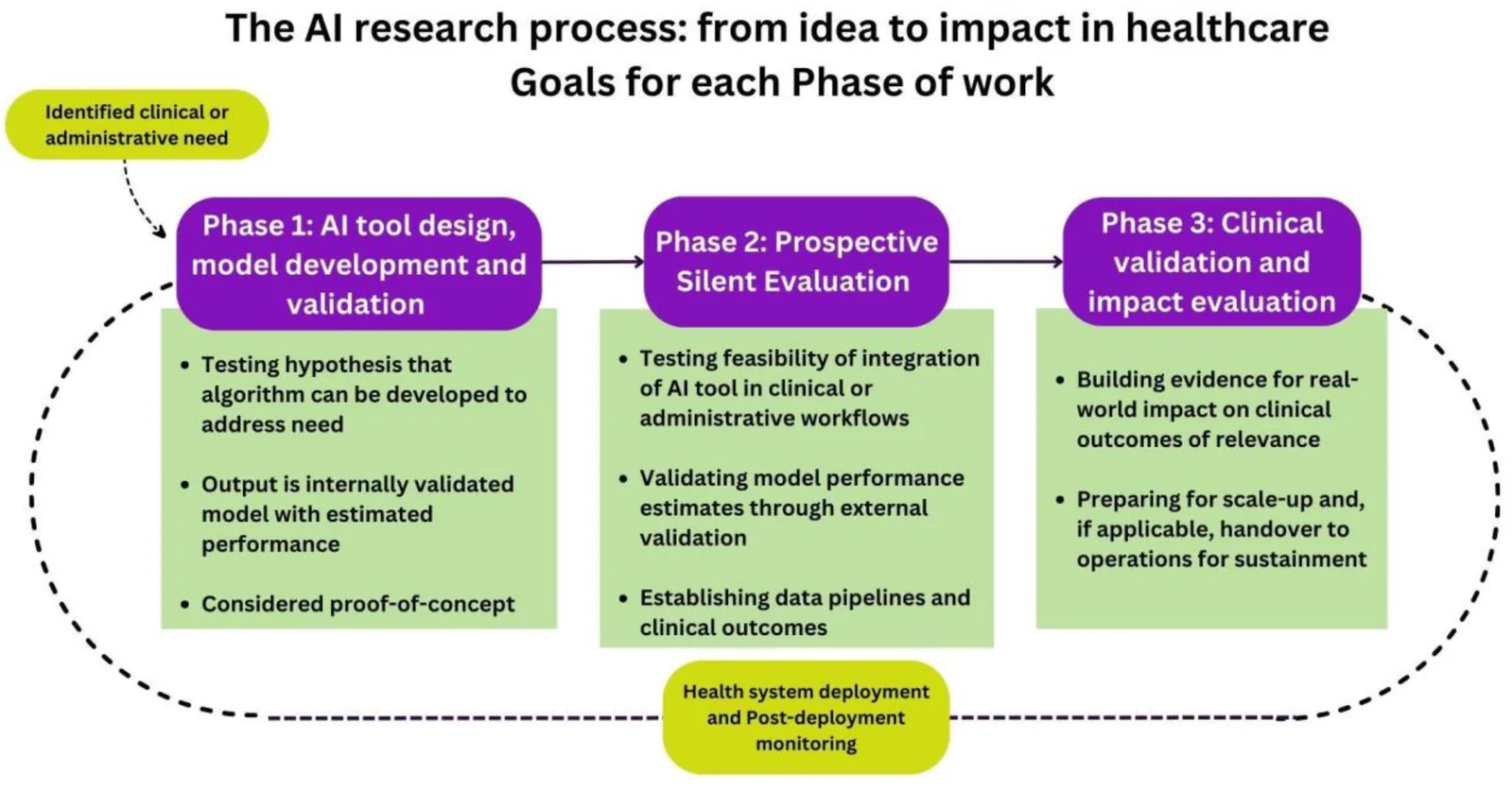
Clinical AI translation pipeline illustrating the sequential pathway from dataset preparation and AI model development through validation, regulatory approval, clinical workflow integration, and ultimate impact on patient outcomes, supported by cross-cutting requirements for trustworthy and sustainable deployment.

The second phase focuses on prospective and external evaluation, where AI models are tested in realistic clinical environments. This stage assesses model generalizability, workflow compatibility, and performance across diverse populations and healthcare settings. External validation is particularly important for determining whether AI systems can maintain reliability beyond the datasets used during development.

The third phase involves clinical validation and impact assessment. Here, evidence is generated regarding the effect of AI-assisted decision support on diagnostic accuracy, screening efficiency, patient management, and healthcare outcomes. Successful completion of this phase provides the foundation for regulatory approval, large-scale implementation, and integration into routine clinical workflows.

Across all phases, several cross-cutting requirements are essential for trustworthy and sustainable deployment, including data quality, reproducibility, transparency, explainability, fairness, privacy protection, regulatory compliance, and continuous post-deployment monitoring. Together, these components support the safe translation of AI innovations from experimental research to clinically effective tools capable of improving breast cancer detection and patient outcomes.

### 8.3 Limitations

While this study provides a comprehensive bibliometric and thematic analysis of AI-based breast cancer research, several important limitations must be acknowledged to ensure a balanced interpretation of the findings.

First, the analysis is inherently constrained by data source bias. The corpus is derived from selected databases and relies primarily on published, indexed studies, which may exclude relevant work available in other repositories, preprints, or non-English publications. Consequently, certain regions, datasets, or research communities may be underrepresented. In addition, the reliance on titles, abstracts, and keywords for quantitative extraction introduces a reporting bias, as not all methodological details—such as datasets, frameworks, or hyperparameter tuning strategies—are consistently documented in these sections.

Second, the findings are limited by a lack of direct clinical validation. Although many studies report high performance metrics (e.g., accuracy, AUC), bibliometric analysis cannot assess whether these models have been validated in real-world clinical environments or integrated into healthcare workflows. As a result, there remains a gap between algorithmic performance and clinical applicability, particularly in areas such as treatment-response prediction and decision-support systems.

Third, issues related to generalizability must be considered. Many studies rely on a small number of widely used benchmark datasets (e.g., CBIS-DDSM, MIAS, INbreast), which may not fully represent diverse patient populations or imaging conditions. This over-reliance can lead to models that perform well on specific datasets but may not generalize effectively across different clinical settings, populations, or modalities. Similarly, the dominance of mammography-based studies may limit insights into underrepresented modalities such as MRI, genomics, and multimodal data integration.

Finally, the study is subject to methodological limitations inherent to bibliometric analysis. Techniques such as citation analysis, keyword co-occurrence, and burst detection capture research activity and trends but do not necessarily reflect true scientific quality, clinical impact, or causal relationships. For example, high citation counts may indicate popularity rather than practical relevance, and keyword-based trend analysis depends on author terminology, which may vary across studies.

Although accuracy is the most reported metric in AI-based breast cancer detection studies, it alone does not adequately reflect clinical usefulness or readiness for real-world implementation. Comprehensive evaluation requires additional performance measures, including sensitivity, specificity, positive predictive value (PPV), negative predictive value (NPV), and the area under the receiver operating characteristic curve (AUC). Sensitivity and specificity are particularly important for balancing cancer detection with false-positive recalls, while PPV and NPV provide insight into the reliability of positive and negative predictions in different screening populations. Although AUC remains a standard measure of discrimination, it should be interpreted alongside these clinically meaningful metrics because models with similar AUC values may differ substantially in their impact on patient management and screening outcomes.

Beyond predictive performance, future research should emphasize calibration and clinical utility to ensure reliable decision-making. Calibration assesses whether predicted probabilities accurately represent the true risk of malignancy, thereby supporting appropriate clinical interpretation. Decision curve analysis (DCA) further complements traditional evaluation metrics by quantifying the net clinical benefit of AI-assisted decisions across different risk thresholds. Ultimately, the value of AI should be determined by its ability to improve early cancer detection, reduce interval cancers and diagnostic delays, increase workflow efficiency, and enhance patient outcomes. Increasing evidence also supports physician–AI collaboration rather than autonomous AI systems, suggesting that future evaluations should compare standalone AI, radiologist-only, and combined physician–AI performance to demonstrate potential synergistic benefits.

Successful clinical translation also depends on seamless integration into healthcare workflows and adherence to regulatory standards. AI systems should be compatible with existing clinical infrastructures, including Picture Archiving and Communication Systems (PACS), radiology information systems, and electronic health records, while providing rapid, user-friendly, and interpretable outputs. At the same time, regulatory approval requires rigorous external validation, transparent reporting, continuous post-market monitoring, and robust risk management strategies. Equally important are fairness and explainability, as AI models must demonstrate consistent performance across diverse patient populations and provide clinically meaningful explanations that enhance physician trust and support informed decision-making.

Finally, widespread adoption of AI in breast cancer detection must be supported by evidence of cost-effectiveness and long-term sustainability. Economic evaluations should consider implementation costs, computational infrastructure, software maintenance, workforce training, and potential reductions in unnecessary imaging, biopsies, and healthcare expenditures. Therefore, future evaluation frameworks should extend beyond traditional accuracy-focused assessments by integrating diagnostic performance, calibration, clinical utility, workflow integration, regulatory readiness, fairness, explainability, and economic impact. Such a multidimensional approach will provide a more realistic assessment of AI readiness for routine clinical practice and facilitate its safe, ethical, and effective integration into breast cancer care.

**Figure 14** illustrates the progressive translation of artificial intelligence from model development to routine clinical practice. The first stage focuses on algorithm performance, emphasizing model development, diagnostic evaluation using metrics such as accuracy, sensitivity, specificity, AUC, calibration, and decision curve analysis, together with model interpretability. The second stage highlights clinical validation, including external and multicenter validation, prospective real-world studies, clinical utility assessment, and regulatory and ethical evaluation. The final stage represents clinical deployment, where validated AI systems are integrated into clinical workflows through physician–AI collaboration, continuous performance monitoring, and workflow integration, ultimately improving diagnostic accuracy, patient outcomes, and healthcare efficiency. Cross-cutting considerations—including data quality, privacy, fairness, explainability, governance, and clinician training—support all stages of the translation pipeline.

**Figure 14:**
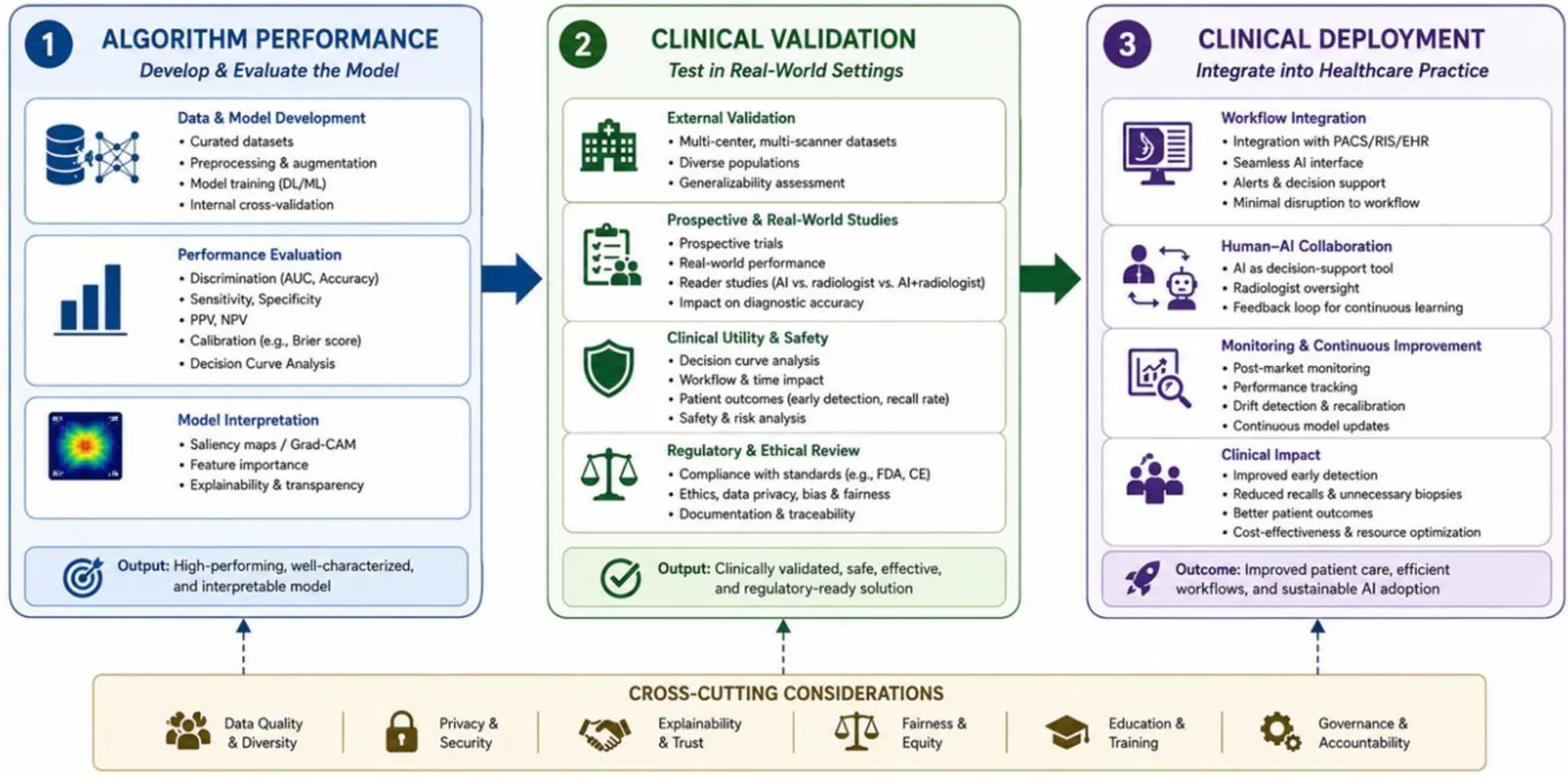
Pathway from algorithm performance to clinical deployment of AI-based breast cancer detection systems.

Overall, these limitations highlight the need for improved reporting standards, broader dataset diversity, rigorous clinical validation, and standardized evaluation frameworks in future research. Addressing these challenges is essential for translating AI-based breast cancer models from experimental settings into reliable, generalizable, and clinically impactful solutions.

### 8.4 Synthesis of bibliometric findings, technological evolution, and future research priorities

The bibliometric findings highlight a rapidly evolving research landscape in AI-based breast cancer detection between 2020 and 2026, characterized by continuous methodological innovation and increasing clinical relevance. The temporal analysis of research themes revealed a clear progression from traditional machine learning and convolutional neural network (CNN)-based approaches toward more sophisticated frameworks involving vision transformers (ViTs), explainable artificial intelligence (XAI), multimodal learning, and advanced imaging modalities such as digital breast tomosynthesis. This evolution reflects the broader transition of artificial intelligence research from achieving high predictive performance toward developing trustworthy, interpretable, and clinically deployable diagnostic systems as shown in **Figure 15**.

**Figure 15:**
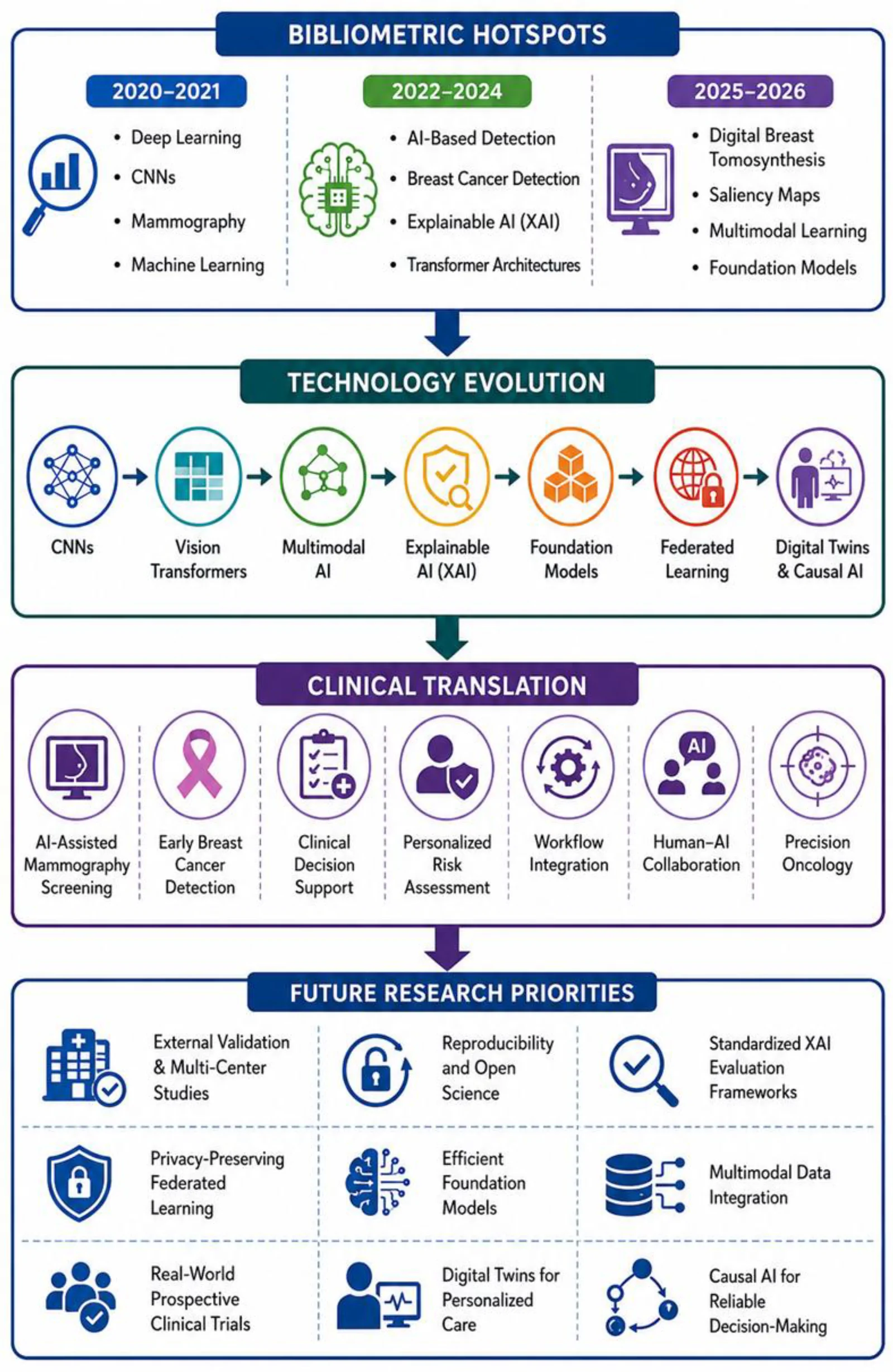
Conceptual framework linking bibliometric hotspots, technological evolution, clinical translation, and future research priorities in AI-based breast cancer detection (2020–2026).

The citation and productivity analyses further indicate that the most influential studies have been those introducing novel deep learning architectures, explainability frameworks, and automated diagnostic systems capable of supporting breast cancer screening and clinical decision-making.

The technological evolution identified through keyword analysis is strongly supported by the increasing availability of large-scale public datasets and advanced computational tools. Clinical registries, imaging repositories, and genomic databases provide the empirical foundation required for training and validating robust AI models, while machine learning and bioinformatics software platforms enable scalable development, evaluation, and deployment. Nevertheless, bibliometric evidence suggests that despite the rapid growth of publications, challenges related to data heterogeneity, reproducibility, external validation, and transparency remain major barriers to clinical implementation. Future studies should therefore prioritize standardized reporting practices, open-access datasets, publicly available code repositories, and comprehensive documentation of training, preprocessing, and evaluation procedures to improve reproducibility and facilitate cross-study comparison.

Several emerging research directions are expected to shape the next generation of AI-assisted breast cancer diagnosis. First, transformer-based architectures require extensive multi-institutional validation to establish their generalizability across diverse patient populations, imaging protocols, and healthcare settings. The development of foundation models, self-supervised learning techniques, and computationally efficient transformer variants represents a promising strategy for reducing dependence on large annotated datasets while supporting real-time clinical deployment. Second, explainable artificial intelligence is anticipated to become a central component of future diagnostic systems. Research efforts should focus on developing clinically meaningful explanation mechanisms, interactive visualization tools, multimodal explanatory interfaces, and workflow-integrated XAI solutions that enhance physician trust and support transparent decision-making.

Another important priority involves the advancement of multimodal and inherently interpretable AI systems. Future breast cancer models should integrate mammographic images with complementary clinical, pathological, and genomic information to generate more comprehensive diagnostic assessments. Rather than relying solely on *post hoc* explainability methods, researchers should explore architectures that embed interpretability directly into model design through attention mechanisms, semantic reasoning frameworks, and medical knowledge integration. Such developments may improve clinician confidence while providing a more comprehensive understanding of disease characteristics.

Generative artificial intelligence also represents a promising area for future investigation. Advanced generative models, including generative adversarial networks (GANs) and diffusion-based approaches, may address data scarcity by producing realistic synthetic images and enabling modality synthesis across mammography, ultrasound, and magnetic resonance imaging. However, rigorous clinical validation will be essential to ensure that synthetic data accurately reflect real-world disease characteristics and do not introduce unintended biases that compromise diagnostic reliability.

Finally, the translation of AI innovations into routine clinical practice requires greater emphasis on prospective real-world studies involving healthcare professionals. Longitudinal evaluations, human-AI collaboration studies, workflow integration assessments, and multicenter clinical trials are necessary to determine the effectiveness, usability, and safety of AI systems under real clinical conditions. Additionally, advances in computational efficiency, including multi-fidelity hyperparameter optimization and resource-aware model design, will be essential for enabling cost-effective deployment in diverse healthcare environments.

Overall, the bibliometric evidence suggests that the future of breast cancer AI research lies at the intersection of large-scale data integration, explainable and trustworthy machine learning, multimodal intelligence, generative modeling, and clinically validated implementation frameworks. Addressing these priorities will accelerate the translation of AI technologies from experimental research to routine clinical practice, ultimately improving early detection, diagnostic accuracy, personalized treatment planning, and patient outcomes in breast cancer care.

Bibliometric hotspots demonstrate the transition from CNN-based mammography analysis toward transformer architectures, explainable AI, multimodal learning, and foundation models. These technological advances facilitate clinical translation through AI-assisted screening, diagnosis, and precision medicine applications, while future research priorities focus on validation, reproducibility, multimodal integration, federated learning, foundation models, digital twins, and causal AI to support trustworthy and clinically deployable breast cancer diagnostic systems as shown in **Figure 15**.

**Table 18** summarizes the current maturity, research momentum, and future prospects of the major AI technologies being applied to breast cancer detection and diagnosis. The findings indicate that convolutional neural networks (CNNs) remain the most clinically mature technology, benefiting from extensive validation and widespread adoption in mammographic image analysis. However, their future growth may be constrained by limited explainability, which has prompted increasing interest in more transparent and interpretable approaches.

**Table 18:** Challenges and readiness of emerging AI technologies for breast cancer.

Technology	Bibliometric growth	Clinical maturity	Main challenges	Future potential
CNN	High	High	Limited explainability	Moderate
Vision Transformer	Very High	Moderate	External validation	Very High
Foundation Models	Emerging	Low	Data, computation	Very High
Federated Learning	Emerging	Low	Privacy, interoperability	High
Multimodal AI	High	Moderate	Data integration	Very High
XAI	High	Moderate	Standardization	High
Digital Twins	Early	Very Low	Clinical evidence	Long-term
Causal AI	Early	Very Low	Methodological maturity	Long-term

Among emerging methodologies, Vision Transformers (ViTs) demonstrate the strongest bibliometric growth and one of the highest future potentials. Their ability to capture long-range relationships within medical images has resulted in superior performance across several imaging tasks. Nevertheless, broader clinical adoption requires rigorous external validation across diverse healthcare settings and patient populations to establish reliability and generalizability. Similarly, multimodal AI shows very high future potential because of its capacity to integrate imaging, clinical, pathological, and genomic information, thereby supporting more comprehensive and personalized diagnostic decision-making. However, challenges related to data harmonization and integration remain significant obstacles.

The table also highlights a new generation of AI paradigms with transformative potential. Foundation models are attracting considerable research attention due to their scalability and ability to learn from large, heterogeneous datasets, although substantial computational requirements and data availability issues currently limit clinical deployment. Federated learning offers an attractive solution for privacy-preserving collaborative model development, particularly in healthcare environments where data sharing is restricted. However, interoperability challenges and differences in institutional infrastructure must be addressed before widespread implementation can occur. Likewise, explainable artificial intelligence (XAI) has become a major research focus because of the growing demand for transparent and trustworthy AI systems, although standardized evaluation frameworks remain lacking.

Finally, digital twins and causal AI represent early-stage technologies with long-term strategic importance. While both currently exhibit very low clinical maturity, they hold promise for advancing precision oncology through personalized disease modeling, treatment simulation, and causal inference-based decision support. Their future clinical impact will depend on methodological advances, stronger empirical evidence, and large-scale validation studies. Overall, the table suggests that the future of breast cancer AI will likely be driven by the convergence of transformer architectures, multimodal learning, explainability, privacy-preserving approaches, and next-generation foundation models, moving the field toward more robust, interpretable, and clinically integrated diagnostic systems.

### 8.5 Future research directions and clinical implications

Breast cancer remains one of the most significant global health challenges, with more than 2.3 million new cases diagnosed annually worldwide and a continuously increasing demand for accurate, scalable, and accessible diagnostic solutions (2). Although artificial intelligence has demonstrated remarkable potential in breast cancer detection, classification, prognosis, and treatment planning, the transition from experimental models to clinically deployed systems remains incomplete. The bibliometric findings of this review indicate that the field has evolved rapidly between 2020 and 2026, progressing from conventional machine learning and convolutional neural network (CNN)-based approaches toward advanced paradigms such as Vision Transformers (ViTs), explainable artificial intelligence (XAI), multimodal learning, foundation models, and privacy-preserving AI. However, continued progress will depend on addressing foundational challenges related to data quality, reproducibility, external validation, clinical integration, and regulatory acceptance.

### 8.6 Data infrastructure, software ecosystems, and open science

The future success of AI-based breast cancer research depends fundamentally on the availability of high-quality datasets and advanced computational infrastructures. As presented in **Table 5**, publicly accessible clinical registries, imaging repositories, and genomic databases provide the empirical foundation necessary for developing, training, and validating AI models across the entire breast cancer care continuum, from screening and diagnosis to prognosis and survival prediction (21–26, 29). These resources enable the creation of large-scale, diverse, and representative datasets that are essential for improving model robustness and reducing population-specific biases.

Equally important are the software ecosystems summarized in **Table 6**, which provide machine learning, deep learning, bioinformatics, and statistical analysis capabilities (27, 28). Modern AI research increasingly requires sophisticated infrastructures capable of handling high-dimensional, multi-modal, and longitudinal healthcare data. Consequently, future investments should emphasize interoperable research platforms, standardized data architectures, and scalable computational environments capable of supporting large-scale collaborative AI development.

The increasing complexity of AI systems also highlights the need for stronger commitments to reproducibility and transparent reporting. Access to datasets should be complemented by detailed documentation of preprocessing procedures, dataset partitions, annotation methodologies, training protocols, hyperparameter configurations, and evaluation strategies. Open-source code repositories pretrained model weights, and externally validated benchmark datasets can substantially improve scientific reproducibility and facilitate meaningful comparison across studies. Future research should therefore prioritize open science practices and standardized reporting guidelines to accelerate clinical translation and improve confidence in published findings.

### 8.7 Foundation models and vision-language AI

Recent bibliometric trends suggest that the field is transitioning toward foundation models and vision-language AI systems capable of learning generalized representations from large and heterogeneous medical datasets. Unlike conventional task-specific models, foundation models can be adapted to multiple downstream applications, including breast cancer detection, lesion characterization, risk assessment, prognosis prediction, and treatment response evaluation. Their flexibility offers substantial advantages for developing scalable and reusable AI systems.

A closely related development is the emergence of self-supervised learning (SSL) approaches. Since expert annotation of mammograms, MRI scans, ultrasound images, and histopathology slides is both time-consuming and expensive, SSL methods can leverage vast quantities of unlabeled medical data to learn clinically relevant representations. By reducing dependence on manually annotated datasets, self-supervised approaches have the potential to improve generalizability while lowering development costs.

The integration of foundation models with vision-language architectures represents another promising direction. These systems combine image analysis with textual information extracted from radiology reports, pathology findings, clinical notes, and electronic health records. Such multimodal reasoning capabilities may ultimately support the development of comprehensive clinical decision-support systems. However, despite their considerable potential, foundation models currently face significant barriers including computational requirements, model interpretability, governance concerns, external validation requirements, and regulatory oversight.

### 8.8 Multimodal learning for precision oncology

Future AI systems are expected to move beyond isolated image analysis toward multimodal learning frameworks that integrate diverse sources of patient information. Breast cancer diagnosis and management are inherently multifactorial processes involving imaging findings, pathological features, genomic alterations, laboratory results, family history, and clinical variables. Consequently, multimodal AI offers the opportunity to generate more comprehensive and clinically meaningful assessments than single-modality approaches.

The growing interest in multimodal learning reflects the broader shift toward precision oncology. Future systems may simultaneously analyze mammograms, ultrasound images, MRI examinations, histopathology slides, genomic profiles, and electronic health records to support individualized risk prediction, diagnosis, prognosis estimation, and treatment planning. Such integrated models could potentially improve diagnostic accuracy while providing a more holistic understanding of disease biology.

Nevertheless, several challenges remain. Data harmonization, modality alignment, missing information, interoperability constraints, and computational complexity continue to hinder the development of robust multimodal systems. Future research should focus on standardized integration frameworks and clinically validated multimodal architectures capable of functioning reliably across different healthcare environments.

### 8.9 Explainable and trustworthy AI

Although recent models have achieved impressive diagnostic performance, clinical implementation requires more than predictive accuracy alone. The bibliometric analysis revealed a rapidly growing emphasis on explainable artificial intelligence (XAI), reflecting increasing recognition that transparency and trust are essential components of clinical adoption.

Current explainability approaches such as saliency maps, Grad-CAM, SHAP, and attention visualizations have improved the transparency of AI predictions; however, these techniques often provide only limited insight into the actual reasoning process of complex models. Future XAI systems should therefore focus on generating explanations that align with clinical workflows and physician decision-making processes. Rather than merely highlighting image regions, next-generation explainability frameworks should integrate visual, textual, and contextual clinical explanations that facilitate human understanding and trust.

Advanced explanatory interfaces may include interactive heatmaps, layered visualization systems, natural-language explanations, conversational AI interfaces, and multimodal explanatory frameworks combining imaging findings with clinical information. Furthermore, explainability should be considered within the broader context of trustworthy AI, encompassing fairness, robustness, transparency, accountability, calibration, and reproducibility. Standardized evaluation frameworks for XAI will be essential to ensure consistent assessment and regulatory acceptance.

### 8.10 Federated learning and privacy-preserving AI

The increasing availability of healthcare data has been accompanied by growing concerns regarding privacy, security, and regulatory compliance. Consequently, federated learning has emerged as an important paradigm for collaborative AI development. Rather than centralizing patient data, federated learning allows institutions to train shared models while keeping sensitive information within local healthcare environments.

This approach offers several advantages. By enabling collaboration across multiple healthcare systems, federated learning can improve model generalizability, reduce institutional bias, and facilitate access to larger and more diverse patient populations. Such capabilities are particularly valuable in breast cancer research, where data heterogeneity and demographic variation significantly influence model performance.

Despite its promise, federated learning remains an emerging technology with several unresolved challenges. Data heterogeneity, communication efficiency, cybersecurity concerns, interoperability barriers, and governance frameworks must be addressed before large-scale implementation becomes feasible. Future research should focus on developing secure, scalable, and standardized federated learning infrastructures capable of supporting global collaborations while maintaining patient privacy and regulatory compliance.

### 8.11 Causal AI and digital twins

While most current AI systems focus on predictive modeling, future research is increasingly exploring causal AI and digital twin technologies as transformative paradigms for precision medicine. Traditional machine learning algorithms are highly effective at identifying correlations but often struggle to distinguish causal relationships from statistical associations. In contrast, causal AI seeks to understand cause-and-effect mechanisms that may better reflect underlying disease biology and clinical reasoning.

In breast cancer care, causal models could support more robust risk assessment, treatment selection, and outcome prediction by identifying factors that genuinely influence disease progression and therapeutic response. Although causal AI remains at an early stage of methodological development, it represents an important step toward clinically interpretable and actionable machine intelligence.

Similarly, digital twins represent one of the most ambitious long-term visions in healthcare AI. Digital twins are virtual patient-specific models constructed from imaging, pathological, genomic, physiological, and clinical data. These systems may eventually simulate disease evolution, predict treatment outcomes, optimize therapeutic strategies, and support precision oncology. Although their current clinical maturity remains low, advances in data integration, computational modeling, foundation models, and multimodal learning may gradually enable their adoption in future healthcare environments.

### 8.12 Human–AI collaboration in clinical decision support

The future role of AI in breast cancer care is increasingly viewed as a model of collaboration rather than replacement. Evidence suggests that AI systems achieve the greatest clinical value when functioning as decision-support tools that augment physician expertise rather than operate independently. Consequently, future development efforts should prioritize human-centered design principles and workflow integration.

Effective physician–AI collaboration requires systems that provide actionable recommendations, transparent reasoning, and intuitive interfaces. AI outputs should be seamlessly integrated into existing radiology and oncology workflows, including Picture Archiving and Communication Systems (PACS), electronic health records, and multidisciplinary tumor boards. User-centered design processes involving radiologists, oncologists, pathologists, and healthcare administrators are essential to ensure practical usability and clinical acceptance.

Additionally, future studies should evaluate not only algorithm performance but also physician–AI interaction, cognitive workload, diagnostic confidence, workflow efficiency, and patient outcomes. These human factors will ultimately determine the real-world value of AI-assisted healthcare systems.

### 8.13 Clinical translation and real-world validation

Despite substantial technological progress, the gap between research performance and routine clinical implementation remains considerable. Many published studies continue to rely on retrospective datasets, single-center cohorts, and limited external validation. Consequently, future research must prioritize prospective clinical studies, multicenter validation, and real-world implementation assessments.

Evaluation should also extend beyond traditional metrics such as accuracy and AUC. Clinically meaningful assessments should incorporate sensitivity, specificity, positive predictive value (PPV), negative predictive value (NPV), calibration, decision curve analysis, clinical utility, cost-effectiveness, fairness, and physician acceptance. Regulatory approval pathways increasingly require evidence of safety, reliability, and real-world benefit in addition to technical performance.

Importantly, clinical adoption timelines are likely to differ across technologies. CNN-based systems and selected mammography screening tools have already achieved relatively high levels of clinical maturity. Vision Transformers and multimodal AI systems may reach broader deployment in the medium term following extensive validation. Foundation models, clinical foundation models, and federated learning frameworks are likely to require additional development and regulatory evaluation, whereas causal AI and digital twin technologies currently represent longer-term research directions. As illustrated in **Table 18**, each technological paradigm exhibits distinct levels of readiness, challenges, and future potential.

The future of breast cancer AI is unlikely to be dominated by a single methodology. Instead, progress will emerge from the convergence of multiple complementary paradigms, including self-supervised learning, foundation models, vision-language AI, multimodal learning, explainable AI, federated learning, causal AI, digital twins, and human-centered clinical decision-support systems. Together, these technologies have the potential to transform breast cancer screening, diagnosis, prognosis, and treatment planning.

However, technological innovation alone will not guarantee clinical impact. The ultimate success of AI in breast cancer care will depend on rigorous external validation, reproducibility, transparency, fairness, privacy preservation, regulatory compliance, workflow integration, and demonstrable improvements in patient outcomes. Supported by the evolving data resources described in **Table 5**, the software infrastructures summarized in **Table 6**, and the technology-readiness framework presented in **Table 18**, future research should focus on developing trustworthy, interoperable, and clinically deployable AI ecosystems capable of advancing precision oncology and improving global breast cancer care.

To provide a concise visual summary of the preceding discussion, **Figure 16** presents a spider chart illustrating the major challenges associated with the clinical translation of AI-based healthcare systems. These challenges include generalizability, explainability, fairness, external validation, workflow integration, regulatory compliance, cost-effectiveness, and reproducibility.

**Figure 16:**
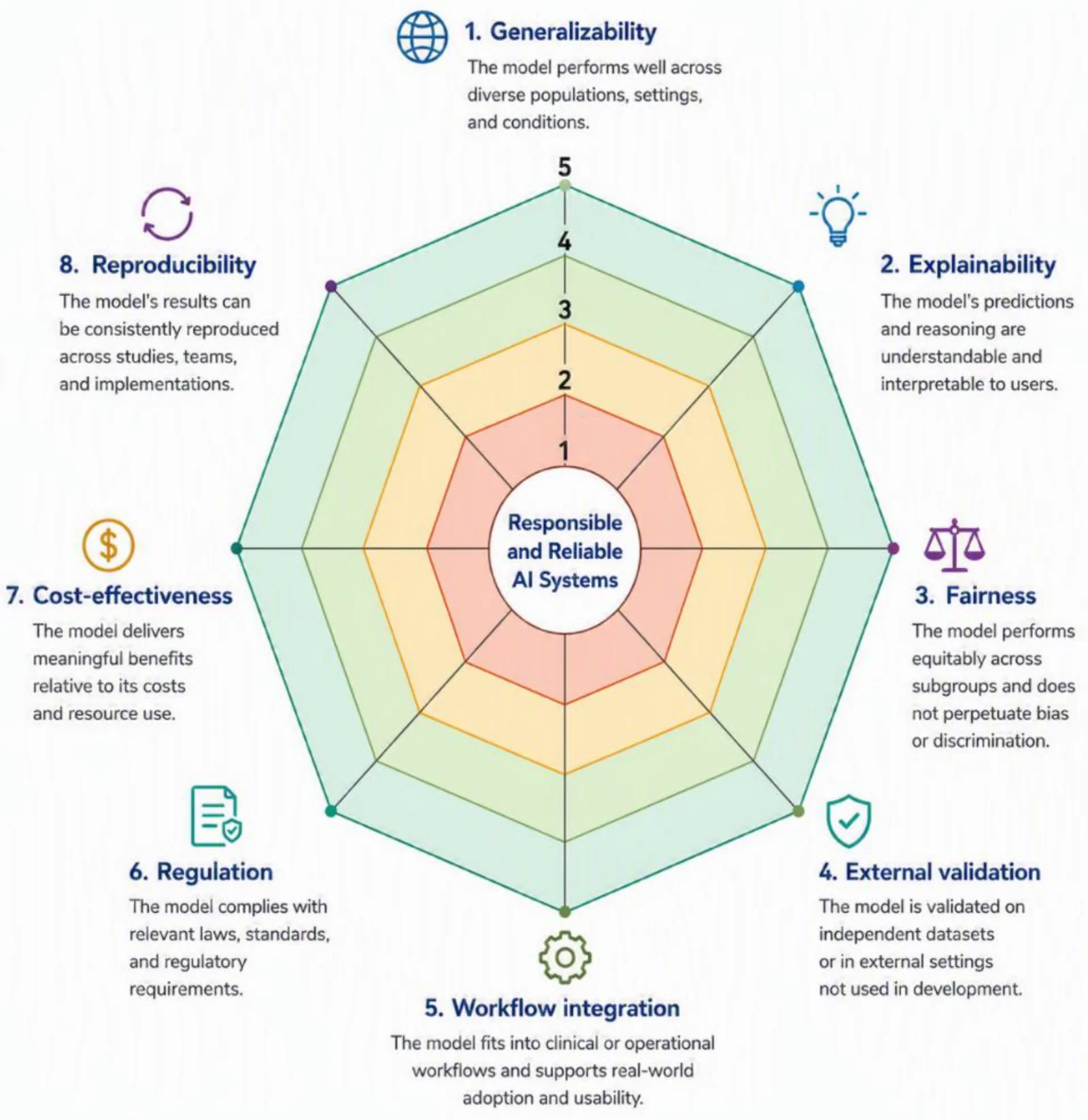
Challenges to the clinical translation of AI-based healthcare systems.

### 8.14 Realistic timelines and remaining barriers to clinical adoption

Despite the remarkable progress of AI in breast cancer detection, widespread clinical adoption is expected to occur gradually over the next 5–10 years, with implementation varying across healthcare systems and resource availability. AI-assisted mammography screening and decision-support systems are already being introduced in several institutions, particularly for image triage, lesion detection, and workflow optimization. However, fully autonomous AI systems that independently guide diagnosis or treatment decisions are unlikely to become routine in the near future. Instead, the most realistic pathway is the continued adoption of human–AI collaborative models, where AI supports rather than replaces radiologists.

Several technical barriers remain before AI systems can be routinely deployed. Many published models are developed using retrospective, single-center datasets and lack robust external validation across diverse populations, imaging devices, and clinical environments. In addition, differences in image acquisition protocols, annotation quality, and disease prevalence limit model generalizability. The limited availability of large, multimodal, and longitudinal datasets also restricts the development of AI systems capable of supporting prognosis prediction and treatment-response assessment.

Clinical and regulatory challenges are equally significant. Successful deployment requires seamless integration with hospital information systems, Picture Archiving and Communication Systems (PACS), and existing radiology workflows without increasing clinician workload. Furthermore, AI systems must satisfy stringent regulatory requirements, demonstrate safety through prospective multicenter clinical trials, and undergo continuous post-deployment monitoring to detect performance degradation over time. Building clinician trust also requires improved explainability, transparent reporting, and standardized evaluation frameworks that extend beyond conventional performance metrics.

Finally, ethical, economic, and organizational factors will strongly influence the pace of adoption. Ensuring fairness across different demographic groups, protecting patient privacy, addressing cybersecurity risks, and establishing clear legal accountability remain essential prerequisites for responsible AI deployment. In parallel, healthcare organizations must demonstrate that AI systems are cost-effective, sustainable, and capable of improving patient outcomes while reducing diagnostic workload and healthcare costs. Addressing these challenges through interdisciplinary collaboration among clinicians, AI researchers, healthcare providers, regulators, and industry partners will be critical to translating promising research into routine clinical practice.

Ultimately, the success of future AI systems in breast cancer care should be measured not solely by predictive accuracy but by their demonstrated clinical impact. Models achieving excellent performance on retrospective datasets may have limited value if they fail to improve patient outcomes, support clinician decision-making, or integrate effectively into healthcare workflows. Therefore, future evaluations should prioritize clinical utility, patient safety, diagnostic efficiency, cost-effectiveness, fairness, physician acceptance, and real-world effectiveness alongside traditional performance metrics. The transition from algorithmic excellence to meaningful clinical benefit represents the critical next phase in the evolution of AI-assisted breast cancer diagnosis and precision oncology.

## Data Availability

The original contributions presented in the study are included in the article/supplementary material. Further inquiries can be directed to the corresponding authors.
